# CRISPR-Cas-amplified urinary biomarkers for multiplexed and portable cancer diagnostics

**DOI:** 10.1038/s41565-023-01372-9

**Published:** 2023-04-24

**Authors:** Liangliang Hao, Renee T. Zhao, Nicole L. Welch, Edward Kah Wei Tan, Qian Zhong, Nour Saida Harzallah, Chayanon Ngambenjawong, Henry Ko, Heather E. Fleming, Pardis C. Sabeti, Sangeeta N. Bhatia

**Affiliations:** 1 Koch Institute for Integrative Cancer Research, Massachusetts Institute of Technology, Cambridge, MA USA; 2 Institute for Medical Engineering and Science, Massachusetts Institute of Technology, Cambridge, MA USA; 3 Broad Institute of Massachusetts Institute of Technology and Harvard, Cambridge, MA USA; 4 Harvard Program in Virology, Division of Medical Sciences, Harvard Medical School, Boston, MA USA; 5 Howard Hughes Medical Institute, Chevy Chase, MD USA; 6 Department of Organismic and Evolutionary Biology, Harvard University, Cambridge, MA USA; 7 Department of Immunology and Infectious Disease, Harvard T.H. Chan School of Public Health, Harvard University, Boston, MA USA; 8 Department of Electrical Engineering and Computer Science, Massachusetts Institute of Technology, Cambridge, MA USA; 9 Department of Medicine, Brigham and Women’s Hospital and Harvard Medical School, Boston, MA USA

**Keywords:** Biosensors, Biomedical engineering

## Abstract

Synthetic biomarkers, bioengineered sensors that generate molecular reporters in diseased microenvironments, represent an emerging paradigm in precision diagnostics. Despite the utility of DNA barcodes as a multiplexing tool, their susceptibility to nucleases in vivo has limited their utility. Here we exploit chemically stabilized nucleic acids to multiplex synthetic biomarkers and produce diagnostic signals in biofluids that can be ‘read out’ via CRISPR nucleases. The strategy relies on microenvironmental endopeptidase to trigger the release of nucleic acid barcodes and polymerase-amplification-free, CRISPR-Cas-mediated barcode detection in unprocessed urine. Our data suggest that DNA-encoded nanosensors can non-invasively detect and differentiate disease states in transplanted and autochthonous murine cancer models. We also demonstrate that CRISPR-Cas amplification can be harnessed to convert the readout to a point-of-care paper diagnostic tool. Finally, we employ a microfluidic platform for densely multiplexed, CRISPR-mediated DNA barcode readout that can potentially evaluate complex human diseases rapidly and guide therapeutic decisions.

## Abstract (web summary)

Protease profiling of the tumour microenvironment using DNA-barcoded nanosensors and CRISPR amplification enables portable cancer diagnosis in preclinical models.

## Main

Biomarkers that provide integrated diagnostic information play essential roles in improving clinical management of complex diseases, ranging from risk assessment, early detection, treatment or surveillance monitoring^1,2^. Since endogenous biomarkers such as shed proteins or nucleic acids can be limited by either their abundance or stability in circulation, engineered ‘synthetic biomarkers’ are in development that leverage the biochemistry of the diseased microenvironment to amplify the production of analytes with improved signal-to-noise ratios^3–7^. Clinical application of molecular diagnostics to classify diverse, dynamic disease states also requires highly multiplexed readouts.

Molecular strategies for multiplexing abound in analytical chemistry, with DNA-barcoding transforming drug screening, gene expression profiling and cellular function analysis^8–10^. However, the susceptibility of nucleic acids in vivo^11^ impedes DNA multiplexing for synthetic biomarkers. Even measurements of endogenous, cell-free nucleic acids are hampered by sequence degradation when not protected by the nucleosome in circulation^12–16^. Therapeutic nucleic acids with chemical modifications can resist systemic degradation, but cannot be amplified with conventional polymerases, or detected in a cost-effective manner^17–19^, preventing their incorporation into diagnostic platforms.

**Fig. 1:**
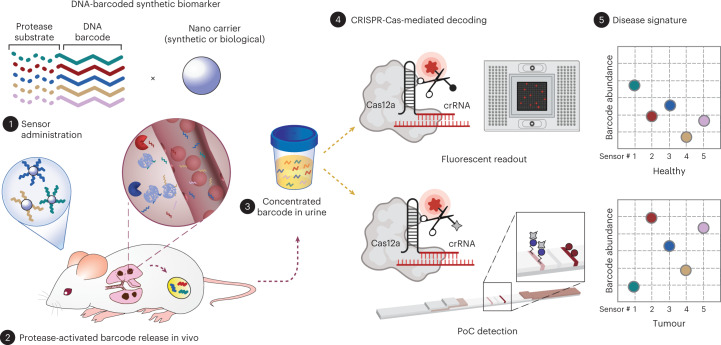
Engineering DNA-encoded synthetic urine biomarkers with CRISPR-Cas-mediated disease detection. DNA-encoded synthetic urine biomarkers are comprised of a nanocarrier (synthetic or biological) functionalized with protease-activated short peptides barcoded with oligonucleotides (1). After in vivo administration, activation of DNA-encoded sensors by disease-specific protease activity triggers release of synthetic DNA barcodes (2) that are size-specifically concentrated in the urine for disease monitoring (3). DNA barcodes in the urine activate programmable CRISPR-Cas nucleases to release the multiplexed reporter signals that are fluorescent, or detected on paper (4), enabling in situ disease classification at the PoC via the patterns of local proteolytic activities in the disease microenvironment (5).

To multiplex synthetic biomarkers for use as point-of-care (PoC) precision diagnostics, we exploited chemically stabilized DNA for molecular barcoding and employed CRISPR-Cas nucleases for a sequence-specific readout^20^. By integrating these enabling technologies, we engineered a panel of high-throughput, programmable in vivo nanosensors for non-invasive disease detection and monitoring in urine (Fig. 1). These rationally designed, DNA-encoded synthetic urine biomarkers (DNA-SUBs) (1) target diseased sites in vivo, (2) sense dysregulated proteolysis in the tumour microenvironment^21,22^, and (3) emit nucleic acid barcodes that survive circulation and are size-concentrated by the kidney^23–25^ to (4) activate single-stranded DNase activity of CRISPR-Cas12a via sequence complementarity to a guide RNA. Liberated by pathological proteolytic activities, chemically stabilized DNA barcodes were detected in unprocessed urine using fluorescence kinetics or on paper strips upon Cas12a activation. The massive multiplexity enabled by DNA barcoding opens the door to classify complex human disease in the context of comorbidities. To demonstrate their modularity, we assembled DNA-SUBs on biological (nanobody) or synthetic (polymer) scaffolds and performed non-invasive detection and monitoring of three different types of primary and metastatic cancers in murine models.

## Chemically modified DNA enables CRISPR-Cas12a-mediated urine test

**Fig. 2:**
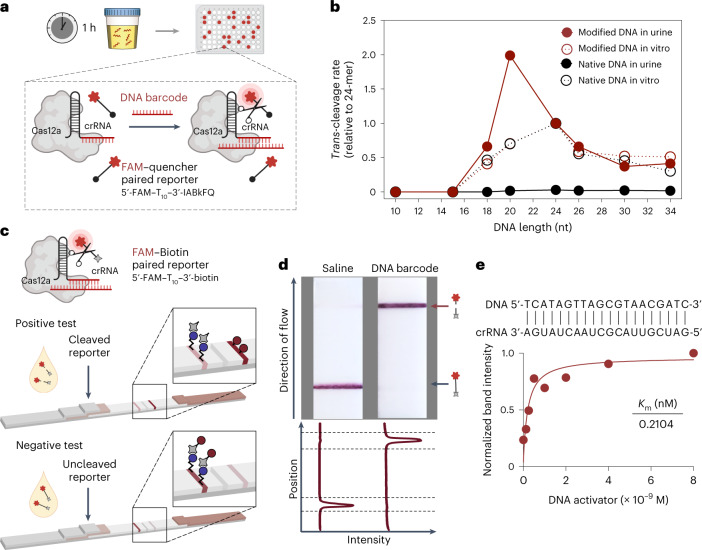
Chemically modified DNA enables CRISPR-Cas-mediated urinary readout for in vivo sensing. **a**, Schematic of urine testing in a murine model and the study time course. Urine samples were collected from mice injected with 1 nmol of ssDNA activator after 1 h of intravenous administration. DNA fragment activates the collateral nuclease activity of Cas12a upon binding to crRNA. Such activity can be tracked by the dequenching of fluorescence from a fluorophore (F, 5′-FAM)–quencher (Q, 3′-IABkFQ) paired oligonucleotide. The initial reaction velocity (*V*_0_) is determined from the slope of the curve at the beginning of a reaction. **b**, Length optimization of ssDNA activator in vitro and in vivo, by quantifying the *trans*-cleavage rate of Cas12a upon activation of native or modified ssDNA in solution (4 nM) or mouse urine (1 nmol per injection). The *trans*-cleavage rates (initial reaction velocity, *V*_0_) were normalized to that of a 24-mer. **c**, Schematic showing set-up of paper-based lateral-flow assay. When Cas12a is activated by the DNA fragments in mouse urine, it cleaves the fluorescein (FAM)–biotin paired oligonucleotide reporter and frees the FAM molecule, which can be detected on the ‘sample band’. Uncleaved reporters are trapped on the ‘control band’ via binding of biotin to streptavidin. **d**, Different bands are visible on paper strips. Band intensities were quantified using ImageJ, and each curve was aligned below the corresponding paper strip. The top peak of the curve shows the freed FAM molecule in cleaved reporter samples, and the bottom peak shows the presence of the uncleaved FAM–biotin reporter. **e**, Michaelis–Menten plot of *Lba*Cas12a-catalysed ssDNA *trans*-cleavage upon a representative DNA–crRNA pairing (complementary sequences are shown) on paper. Data were plotted with the quantified band intensity of cleaved reporter on paper strips. Km is determined as the DNA activator concentration at which the reaction rate is half of its maximal value. Source data

CRISPR-Cas nucleases may enable DNA and RNA diagnostics^20,26–30^, yet remain untapped for in vivo disease sensing due to degradation of unmodified nucleic acids in circulation. To prevent the susceptibility and immunostimulatory activity of unmodified nucleic acids in vivo, we activated the Cas effector protein CRISPR-Cas12a with FDA-approved phosphorothioate-modified DNA^31–34^. These DNA activators unleash the collateral cleavage activity of Cas12a upon CRISPR RNA (crRNA) binding, leading to *trans*-cleavage of bystander DNA molecules visualized by a fluorescent reporter (Fig. 2a). Specifically, *Lachnospiraceae bacterium* Cas12a (*Lba*Cas12a) recognizes double-stranded DNA (dsDNA) through a T-nucleotide-rich protospacer-adjacent motif (PAM), and single-stranded DNA (ssDNA) in a PAM-independent manner^20^. We observed that phosphorothioated ssDNA does activate *Lba*Cas12a, albeit at a reduced speed relative to native DNA (Supplementary Fig. 1a). When native ssDNA was intravenously administered, urine samples could not activate *Lba*Cas12a assembled with the corresponding crRNA, probably due to circulating, unspecific DNase activity (Fig. 2b and Supplementary Fig. 1b). In contrast, unprocessed urine from animals injected with phosphorothioated ssDNAs retained the ability to trigger the collateral nuclease activity of *Lba*Cas12a (Fig. 2b). Notably, we observed length-dependent changes of Cas12a activation by chemically stabilized ssDNAs. In solution, the highest in vitro cleavage signal was triggered by the 24-mer PAM-containing ssDNA, whereas in vivo, urine samples from mice administered 20-mer PAM-free, crRNA-complementary ssDNA exhibited optimal cleavage activity, highlighting that efficient renal clearance requires size-dependent kidney glomerular filtration^23^ (Fig. 2b and Supplementary Tables 1 and 2). Modified DNA molecules in unprocessed urine were also detectable using a colorimetric readout on lateral-flow paper strips (Fig. 2c). The presence of the leading ‘sample band’ indicated that detectable FAM had been freed via reporter cleavage after *Lba*Cas12a was activated by the urinary DNA (Fig. 2d). In addition to the binary DNA activator detection, we assessed enzyme activation kinetics by quantifying the intensity of the sample bands (Fig. 2e), and assay optimization achieved subnanomolar sensitivity to DNA activators for both fluorescent (Supplementary Fig. 2) and paper-based readouts (Supplementary Fig. 3).

## Tumour-targeting, singleplex DNA-encoded synthetic biomarker

**Fig. 3:**
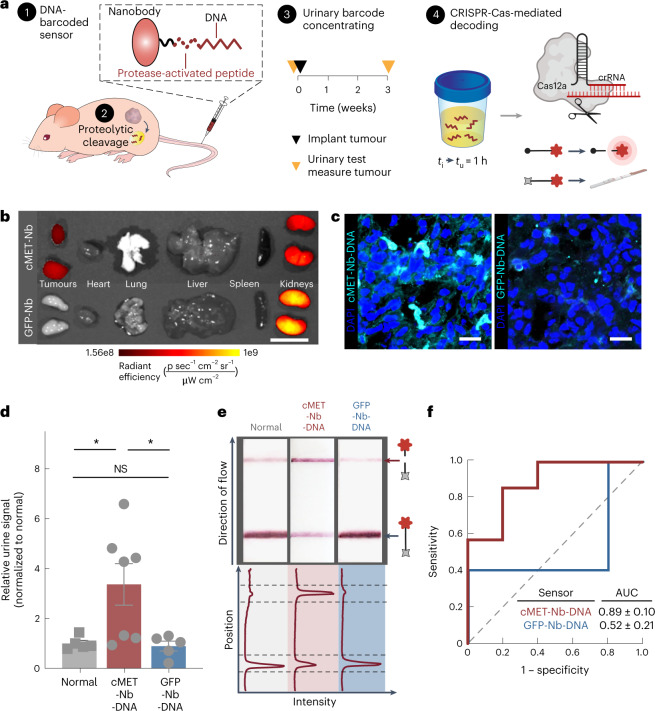
Tumour-targeting, singleplex DNA-encoded synthetic urine biomarker for detection of prostate cancer. **a**, Schematic showing generation of the DNA-encoded protease-activatable nanobody (1). Disease-specific protease activity triggers (2) release of ssDNA activator into urine for disease detection (3). Study time course of urine testing and detection of the ssDNA activator with Cas12a *trans*-cleavage assay using fluorescent or paper readout (4). *t*_*i*_, time of sensor injection; *t*_*u*_, time of urine collection. **b**, IVIS image shows biodistribution of cMET-targeting nanobody and non-targeting GFP nanobody when injected intravenously in nude mice bearing PC-3 xenografts. Colour intensity reflects the radiant efficiency of fluorescence (*λ*_ex_ = 640 nm, *λ*_em_ = 680 nm). Scale bar, 1 cm. **c**, Immunofluorescent staining of Cy7-labelled, DNA-encoded cMET or GFP nanobody on sections of PC-3 tumours. DAPI, 4,6-diamidino-2-phenylindole. Scale bar, 20 µm. **d**, Unprocessed urine samples collected from tumour-bearing mice injected with DNA-encoded cMET or GFP nanobody, and healthy control mice injected with DNA-encoded cMET nanobody were applied in the Cas12a *trans*-cleavage assay. Initial reaction velocities (*V*_0_) were calculated and normalized to that of healthy control mice. *n* = 5 for control groups, *n* = 7 mice for DNA-encoded cMET nanobody injected group; mean ± s.e.m.; one-way analysis of variance with Tukey’s multiple-comparison test, *P = 0.028 for the normal versus cMET-Nb-DNA comparison, *P = 0.0221 for the cMET-Nb-DNA versus GFP-Nb-DNA comparison; NS, not significant where *P* = 0.9902 for the normal versus GFP-Nb-DNA comparison. **e**, Paper-based lateral flow assay (LFA) of Cas12a activation by urine samples collected from tumour-bearing or healthy control mice in **c**. Band intensities were quantified using ImageJ and each curve was aligned below the corresponding paper strip. The top peak of the curve shows the presence of the cleaved reporter and the bottom peak shows the presence of the uncleaved reporter. **f**, ROC curves characterize the predictive power of cMET-Nb-DNA and GFP-Nb-DNA in **c**. Dashed line represents the baseline AUC of 0.5, representing a random biomarker classifier; the AUC of a perfect biomarker is 1.0. Source data

To engineer DNA-encoded synthetic biomarkers for in vivo disease sensing, we leveraged dysregulated proteolytic activities in the tumour microenvironment to cleave and release chemically stabilized DNA barcodes that were concentrated in the urine as a function of their size to produce a non-invasive readout of the target disease. We first evaluated a singleplex synthetic biomarker in a human prostate cancer (PCa) xenograft model^35^. To reduce non-specific sensor release, we engineered the DNA-SUB on a biological scaffold with tumour-targeting capacity. Specifically, we utilized the robust stability and tissue affinity of single-domain antibody fragments (nanobodies) by inserting a peptide substrate sequence with an unpaired cysteine for one-step site-specific labelling of cargoes via a thioether^36,37^ (Fig. 3a, Supplementary Table 3 and Supplementary Fig. 4a,b). The peptide substrate specifically responded to the PCa-associated protease, PLAU^35^ (or uPA, Supplementary Fig. 7e). To prevent possible misfolding caused by the nanobody’s internal disulfide bond, a rigid linker distanced the cysteine-containing peptide substrate from the nanobody scaffold (Supplementary Table 3). For in vivo validation, we tested the PLAU-activated, cMET-targeting nanobody in the cMET- and PLAU-expressing PC-3-cell-derived tumour model (Supplementary Fig. 4c,d). In subcutaneous PC-3 tumours, the cMET nanobody mediated active tumour trafficking upon systemic administration (Fig. 3b). We then covalently linked PLAU-activated nanobody with the 20-mer DNA barcode via click chemistry, and the conjugated nanobody (cMET-Nb-DNA) exhibited enhanced tumour accumulation compared with the DNA-barcoded, PLAU-activated, non-targeting green fluorescent protein (GFP) nanobody (GFP-Nb-DNA)^38^ (Fig. 3c). We systemically administered cMET-Nb-DNA to tumour-bearing and healthy control mice and quantified subsequent urinary DNA barcodes after 1 h. Urine samples were incubated with LbaCas12a-coupled with the complementary crRNA, and the *trans*-cleavage activity triggered by the DNA barcode was analysed by tracking the kinetics of cleavage of a fluorescence-quencher-labelled poly(T) reporter. Administration of cMET-Nb-DNA significantly increased the *trans*-cleavage rate of *Lba*Cas12a activated by urine samples collected from tumour-bearing mice relative to healthy controls (Fig. 3d).

To translate the fluorescent readout into a PoC detection tool, we incubated *Lba*Cas12a activated by mouse urine samples with the FAM-poly(T)–biotin reporter and ran the cleavage products on lateral-flow paper strips. An enhanced sample band appeared in samples collected from tumour-bearing mice injected with cMET-Nb-DNA (Fig. 3e). The high sensitivity and specificity of the sensor to track disease was reflected in a receiver operating characteristic (ROC) curve analysis (area under the curve (AUC) = 0.89 ± 0.10, *P* = 0.03). In contrast, urine samples collected from tumour-bearing mice injected with GFP-Nb-DNA activated *Lba*Cas12a at a rate that was almost identical to the samples from healthy controls, indicating that the release of DNA barcodes was triggered on-target (Fig. 3d,e and Supplementary Fig. 4e). This urinary diagnostic robustly detected PC-3-cell-derived tumours that are prostate-specific antigen (PSA) negative (Supplementary Fig. 4f), suggesting broad utility of synthetic urine biomarkers in tumour types without available clinical blood biomarkers.

## Multiplex DNA-encoded synthetic biomarkers in portable diagnostics

**Fig. 4:**
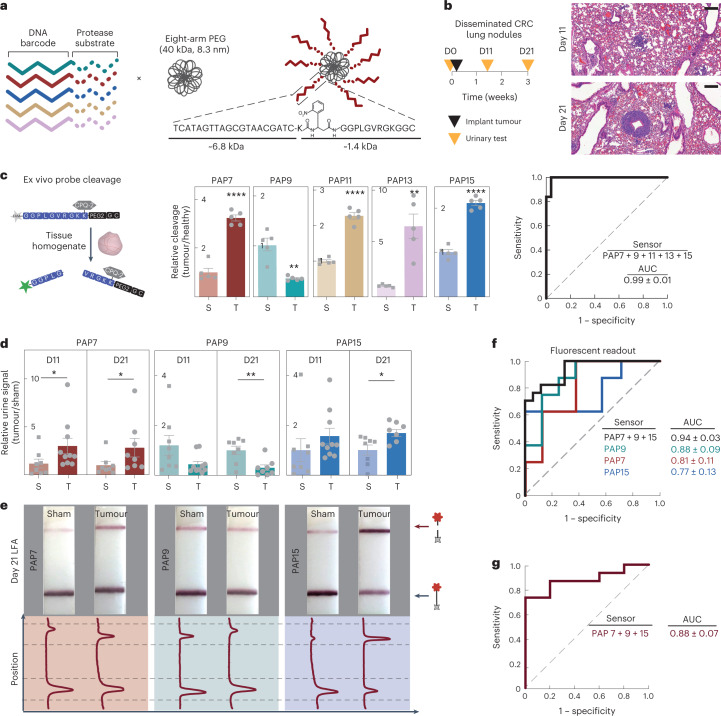
Multiplexed DNA-encoded synthetic urine biomarkers for portable monitoring of invasive colorectal cancer. **a**, Multiplexed DNA-SUBs are comprised of a polymeric nanocarrier (eight-arm PEG) functionalized with protease-activated peptides barcoded with oligonucleotides. **b**, Left: Timeline of longitudinal tumour monitoring with DNA-SUB. D, day. Right: histological lung staining of BALB/c mice bearing CRC lung tumours at 11 days (upper) and 21 days (lower) after tumour inoculation. Scale bar, 200 µm. **c**, Left: Schematic of the fluorogenic assay to identify peptide substrates specifically cleaved by lung tissue homogenates collected 21 days after tumour inoculation. Middle: Peptide cleavage by CRC-bearing and healthy lung tissue homogenates were monitored and cleavage rates normalized to healthy tissue are shown in bar graphs (*n* = 5 mice per group; mean ± s.e.m.; unpaired two-tailed *t*-test with Welch’s correction, ****P < 0.0001 for PAP7, **P = 0.005 for PAP9, ****P < 0.0001 for PAP11, **P = 0.008 for PAP13, ****P < 0.0001 for PAP15). Right: ROC analysis of data in the middle panel. **d**, Pooled DNA-SUBs were administered to CRC lung tumour-bearing BALB/c mice (tumour, T) and saline-injected control animals (sham, S) at day 11 or 21 after tumour initiation. Urine samples were collected at 1 h after sensor administration. Cas12a *trans*-cleavage assays were performed against each DNA barcode and the initial reaction velocities (*V*_0_) were calculated and normalized to those of saline-injected control animals (day 11, n = 10 mice per tumour group; day 21, n = 8 mice per tumour group; n = 8 mice per control group; mean ± s.e.m.; unpaired two-tailed Mann–Whitney test, day 11 *P = 0.0343 for PAP7; day 21 *P = 0.0379 for PAP7; unpaired two-tailed *t*-test with Welch’s correction, **P = 0.0054 for PAP9; *P = 0.0171 for PAP15). **e**, Representative paper readout of Cas12a activation by urine samples collected in **d**. Band intensities were quantified using ImageJ. The top peak of the curve shows cleaved FAM reporter and the bottom peak shows uncleaved FAM–biotin reporter. **f**, ROC analysis showing the ability of individual or combinational DNA-SUBs to distinguish diseased mice and healthy controls with fluorescent readout in **d**. **g**, With the paper readout in **e**, the ROC analysis utilized the ratio of quantified cleaved reporter band over its corresponding control band. Dashed line represents an AUC of a random biomarker classifier (0.5); the AUC of a perfect biomarker is 1.0. Source data

Diagnostic sensitivity and specificity in heterogeneous diseases will probably require analysis of multiple cancer hallmarks. Whereas active targeting is limited to diseases that express specific ligands, multiplexing of an untargeted scaffold has the potential to be more generalizable. Therefore, we constructed a multiplexed panel of DNA-SUBs on a polymer-based scaffold and administered the pooled sensors to mice (Fig. 4a). Each DNA-SUB bore 20-mer phosphorothioated DNA-tagged, protease-activated peptides (PAPs) covalently conjugated to a 40 kDa eight-arm poly(ethylene glycol) (PEG) nanocarrier, which prolonged the blood half-life of cargo (Fig. 4a, Supplementary Fig. 5 and Supplementary Table 3). Upon purification, the completed nanosensors showed increased hydrodynamic diameter, monodispersity and uniform spherical shape (Supplementary Fig. 5a–d).

To monitor nanosensor trafficking to tissue with intact tumour-immune composition, we established a syngeneic mouse model by intravenous administration of MC26-LucF, metastatic murine colorectal cancer (CRC) cells, into immunocompetent BALB/c mice^39^ (Fig. 4b and Supplementary Fig. 6). We sought CRC-specific proteases via transcriptomic analysis and selected those expressed in tumours at >1.5-fold levels over normal samples, including members of the matrix metalloproteinase (MMPs), aspartic and serine protease families (for example, cathepsins, kallikrein-related peptidases) (Supplementary Fig. 7a). Matrisome proteomic analysis confirmed protease expression in primary CRCs and their distant metastases (for example, MMP-7, MMP-9, Cathepsin D, PLAU)^40,41^ (Supplementary Fig. 7c). These proteases were overexpressed in MC26 tumour-bearing lung tissue relative to normal lung tissue (Supplementary Fig. 7b,d). To identify peptide substrates susceptible to the selected proteases, we screened 16 peptide sequences for cleavage by purified recombinant proteases and identified the top five substrates using a fluorogenic activity assay^4,35,42^ (Supplementary Fig. 7e). These protease-activated peptides (PAP7, PAP9, PAP11, PAP13, PAP15) broadly cover metallo, serine and aspartic protease activities (Supplementary Fig. 7e), and were specifically cleaved by tumour tissue homogenates ex vivo with highly predicted disease classification power, and thus were incorporated into the panel of DNA-SUBs for in vivo validation (Fig. 4c and Supplementary Figs. 8 and 9a). A DNA-barcoded, MMP-responsive SUB (DNA-PAP7-SUB) accumulated in CRC lung tumour nodules following intravenous injection (Fig. 4c and Supplementary Fig. 9b). Subsequently, the 5-plex DNA-SUBs were administered to control and CRC-bearing mice over the course of tumour development, and urinary DNA barcodes freed from the nanosensors were quantified 1 h after injection. Urine samples were incubated with *Lba*Cas12a-coupled with five different complementary crRNAs in multiple wells, and the *trans*-cleavage activity triggered by each DNA barcode was analysed by tracking the cleavage kinetics of a fluorescence-quencher-labelled reporter. We found that the MMP-responsive sensor (DNA-PAP7-SUB) succeeded in distinguishing tumour-bearing mice from healthy mice only 11 days after tumour inoculation when the tumour nodules were 1–2 mm (Fig. 4d and Supplementary Fig. 6a). Some sensor differences were amplified over time (Fig. 4d and Supplementary Fig. 9c), and these sensors (DNA-PAP9-SUB, DNA-PAP15-SUB) corresponded to peptides cleaved by serine and metalloproteases in vitro, and also produced distinct cleavage patterns when incubated with homogenates from either tumour-bearing or healthy lung tissues (Fig. 4c and Supplementary Fig. 7e). Based on the ROC curve analysis, the sum of the metallo- (PAP7, PAP15) and serine (PAP9) protease substrate signals significantly increased the classification power of the DNA-SUBs (Fig. 4f). We then combined the 5-plex sensor panel with lateral-flow detection for a visual readout that could enable PoC diagnostics. Using the same urine samples assayed above (Fig. 4d), we designed the lateral-flow assay to read the cleavage of the FAM-poly(T)-biotin reporter at the optimized end time point. Consistent with the fluorescent readout results, the test paper ‘fingerprints’ revealed distinctive Cas12a activation band intensities between tumour-bearing mice and healthy mice (Fig. 4e). Notably, quantification of the band intensity patterns exhibited disease classification power with multiple sensors (Fig. 4g).

## DNA-enabled multiplexity for clinically relevant diagnostics

**Fig. 5:**
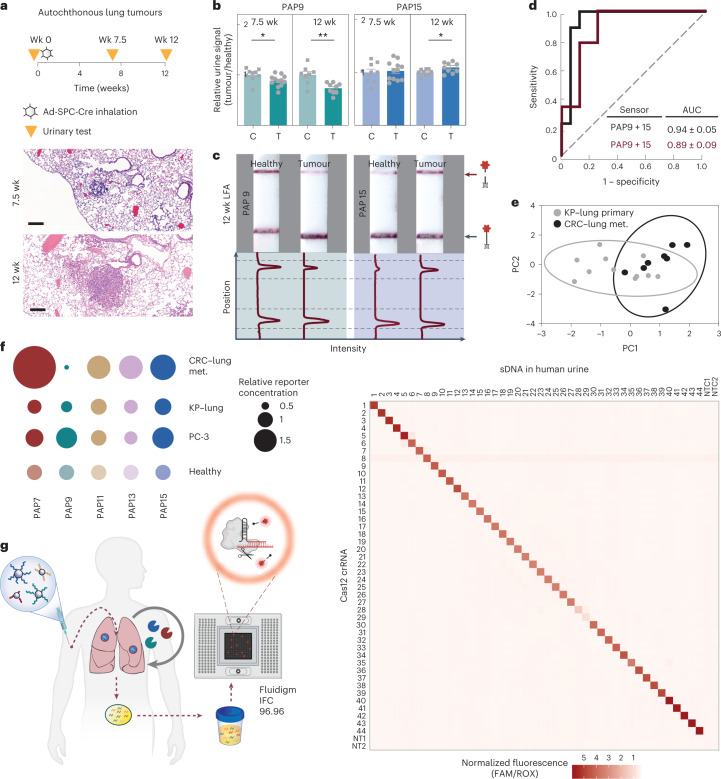
Specificity of the multiplexed synthetic urine biomarkers enabling clinical translation via DNA-barcoding strategy. **a**, Top: scheme of an immunocompetent, autochthonous KP mouse lung cancer model; bottom: histological display of disease progression showing lung tumour nodules at 7.5 and 12 weeks of lentiviral inoculation of Cre recombinase. Wk, week. Scale bar, 200 µm. **b**, Pooled DNA-SUBs were administered to KP (tumour, T) and healthy control B6 mice (control, C) at 7.5 and 12 weeks after tumour initiation. Urine samples were collected at 1 h after sensor administration and Cas12a *trans*-cleavage assays were performed against each DNA barcode with the fluorophore-quencher paired reporter (at 7.5 weeks, *n* = 12 mice per tumour group; *n* = 9 mice per control group; mean ± s.e.m. unpaired two-tailed *t*-test with Welch’s correction, *P = 0.044 for PAP9; at 12 weeks, *n* = 9 mice per tumour group; *n* = 8 mice per control group; mean ± s.e.m., unpaired two-tailed *t*-test with Welch’s correction, **P = 0.004 for PAP9, *P = 0.045 for PAP15). The initial reaction velocity (*V*_0_) refers to the slope of the curve at the beginning of a reaction. **c**, Representative LFA paper strips of Cas12a activation by mouse urine samples collected in **b**. Band intensities were quantified using ImageJ. The top peak of the curve shows the freed FAM molecule in cleaved reporter and the bottom peak shows the presence of the uncleaved FAM–biotin reporter. **d**, ROC curves showing the ability of DNA-SUBs to distinguish KP lung tumour-bearing mice and healthy controls. Black line, fluorescent readout; dark red line, paper readout. Dashed line, AUC of a random biomarker classifier (0.5); the AUC of a perfect biomarker is 1.0. **e**, PCA to reveal the divergence between lung tumour nodules derived from invasive CRC or primary KP mutants. met., metastasis. **f**, The relative fold change between disease states was calculated using mean-scaled reporter concentrations normalized to the corresponding healthy controls. Each dot represents one reporter, and size of dots represents the relative fold change (healthy control = 1). **g**, Left: schematic of massively in-parallel CRISPR-Cas-mediated DNA detection on the Fluidigm microfluidics platform; right: heatmap of *trans*-cleavage rates of different modified ssDNA activator-crRNA pairs. Assays were performed with ssDNA-spiked human urine samples on a Fluidigm microfluidic platform. Source data

Existing endogenous circulating biomarkers (for example, CEA, CA125) suffer from low sensitivity and specificity and do not respond to kinetic changes in tissue microenvironments. We hypothesized that multiplexed measurements of disease-localized protease activity would classify tumours derived from different tissues of origin. In the subcutaneously transplanted PC-3 model, the multiplexed DNA-SUB panel delivered suboptimal disease classification power for PCa (Supplementary Fig. 10), probably reflecting tumour-associated protease activity differences between CRC lung nodules. We next examined whether multiplexed DNA-SUBs could distinguish between tumour types in a single-tissue microenvironment, the lung, by comparing the syngeneic CRC lung transplants and an immunocompetent, autochthonous mouse model of lung adenocarcinoma. Tumours were initiated using intratracheal administration of a virus encoding Cre recombinase to activate mutant *Kras*^G12D^ and delete both copies of Trp53 in the lungs (*Kras*^LSL−G12D/+^;*Trp53*^fl/fl^) (Fig. 5a). This well-characterized KP model histologically recapitulates human disease progression from alveolar adenomatous hyperplasia (AAH) to grade IV adenocarcinoma, reflected by disease progression from low-grade dysplasia to invasive adenocarcinoma over 18–20 weeks^43^. In low-grade disease (7.5 weeks after induction, Fig. 5b), the serine-responsive sensor (DNA-PAP9-SUB) from the multiplexed DNA-SUB panel identified tumour-bearing mice from healthy mice (Fig. 5b and Supplementary Fig. 9d). When disease progressed (12 weeks post-initiation, Fig. 5a), the protease cleavage trend became significant based on a broad MMP-responsive sensor (DNA-PAP15-SUB) in addition to the serine protease-responsive sensor (DNA-PAP9-SUB), reflected in both fluorescent and paper-based barcode readouts (Fig. 5b,c and Supplementary Fig. 9d), allowing for classification of diseased versus healthy control animals in the ROC curve analyses (Fig. 5d).

We then analysed normalized urinary DNA barcode levels to assess whether the panel of DNA-SUBs was differentially cleaved between disease states. Principal-component analysis (PCA) revealed a divergence between the primary KP lung tumours and the disseminated CRC lung nodules (Fig. 5e). We further examined the relative differences in reporter concentrations in diseased and healthy mice across PCa, primary lung and metastatic CRC tumours and found distinct disease signatures, highlighting differential protease activity among tumour types (Fig. 5f). Notably, more sensors were significantly differentially cleaved in the metastatic CRC tumours. Of the two sensors significantly enriched in the urine of mice bearing CRC lung metastasis compared to controls (PAP7, PAP15), only the more generic reporter (PAP15) was also enriched in the KP lung tumour-bearing mice. One additional sensor, PAP9, was also differentially cleaved, even though it was enriched in the healthy controls relative to both lung nodules but not in the PCa model. Collectively, these observations indicated the multiplexed DNA-SUB panel optimized for invasive CRC lung nodules can also detect distinct tumours within the same tissue microenvironment.

Nonetheless, while a 5-plex DNA-SUB panel was sufficient to unveil multiple disease signatures in mice, given the wide range of subtle differences in disease-associated protease activities present in individuals with varied comorbidities, human disease ‘fingerprints’ will most probably require a wider panel of multiplexed DNA-SUBs. To this end, we implemented a massively multiplexed Fluidigm microfluidic platform to maximize the capacity of CRISPR-mediated, diagnostic DNA decoding of protease activities^44^ (Fig. 5g). We evaluated 44 synthetic 20-mer ssDNAs and their corresponding crRNAs in pairwise matching reactions for a total of 2,116 tests (46 × 46, 2 non-targeting pairs included). We observed selective detection of 20-mer ssDNAs both in solution and in human urine samples, with a median AUC of 0.99 across all 44 base-paired crRNAs, with no observed cross-reactivity between different sequences over a defined threshold (6 × s.d. above background), allowing for parallel readout in multiple well assays (Fig. 5g and Supplementary Figs. 11 and 12).

## Discussion

Non-invasive tumour analysis using biomarkers that circulate in biofluids such as blood (‘liquid biopsy’) is reshaping the cancer diagnostics landscape. Our work leverages an emerging, complementary approach, dubbed ‘synthetic biomarkers’^3^, to manufacture molecular signals that overcome current limitations of endogenous biomarkers, such as circulating cell-free DNA. These exogenously administered sensors harness cancer hallmarks (for example, enzymatic activities) for signal amplification, use tumour-selective activation to enhance specificity, and leverage physical biodistribution patterns of nanomaterials in vivo (for example, tumour accumulation, renal clearance) for easy detection. We report an integrated synthetic biomarker platform that enables multiplexed in vivo sensing of microenvironmental proteolytic activity, encodes sensors via DNA barcode intermediates, concentrates via renal filtration, and achieves CRISPR-based amplification to readout on paper. We employ this system in three murine cancer models and demonstrate disease classification with 5-plexed probe sets. We further extend multiplexity by two orders of magnitude (46 × 46) using a microfluidic readout that enables parallelized barcode detection from human urine. The integrated platform fulfils several characteristics of an ideal biomarker: high signal-to-noise ratio, stability in circulation until detection, easy accessibility from host biofluids, and high sensitivity and specificity in disease discrimination.

In the synthetic biomarker platform, diagnostic signals are triggered by disease-associated protease activity. Activity sensing in vivo is typically limited by lack of substrate selectivity due to background cleavage of proteases in normal tissues. We explored two approaches to increase tumour-selective proteolytic activation: tumour localization via affinity targeting, and multiplexing a set of probes to classify disease states. For the former, a protease-activatable cancer-targeting nanobody achieved singleplex diagnosis. For latter, we applied multiplexed sensor panels in both syngeneic and transgenic mouse models to classify diseases based on proteolytic contributions from immune cells within the same lung microenvironment. We observed preferential cleavage of metalloprotease-responsive substrates (PAP15) and a serine protease-sensitive substrate (PAP9) that was reported in the lung^42^. This pattern could point to an imbalance of serine protease/protease inhibitors in the pulmonary environment^45–48^. Notably, when the same multiplexed probe set was used, distinct probe subsets classified other disease types and states, consistent with previous reports of activity-based synthetic biomarkers discriminating primary tumours from benign inflammation, local versus aggressive prostate cancers, and liver fibrosis progression from regression^4,35,42^. Although combinations of two or three probes was sufficient to distinguish disease in isogenic murine tumour models, a densely multiplexed cocktail would probably be necessary to achieve sufficient selectivity to monitor diseases in a heterogeneous patient population. Given the proteolytic network differences in mouse models and human diseases, clinical translation will require additional work including an evaluation of sensor performance in the context of additional comorbidity-bearing animal models, and in human samples.

Urinary detection of cancers in mice via multiplexed synthetic biomarkers showed improved sensitivity relative to existing and emerging blood-based diagnostics^4,24,49^. In an LS174T CRC xenograft model, mass-encoded synthetic biomarkers detect 130 mm tumours^3^, whereas circulating tumour DNA (ctDNA) can detect tumour sizes of >1,000 mm^3^, and carcinoembryonic antigen detection has a threshold of 135–330 mm^3^ (refs. ^4,7,24^). Mass-encoded multiplex sensors are powerful, yet readout by mass spectrometry requires sophisticated instrumentation and extensive data interpretation. Here, through DNA-barcoding, our platform enabled biofluidic cancer readout via cost-effective, CRISPR-mediated lateral-flow assays on paper^27,28,50^. Due to the inherent programmability of Cas proteins, 46 orthogonal barcodes can be readout on a densely multiplexed microfluidic chip (Fig. 5g), a throughput capacity that surpasses commercial isobaric tags for mass spectrometry^4^, and opens the door to assess complex human pathology and identify essential disease classifiers via machine-learning tools.

Despite the lower melting temperature of duplexes, the phosphorothioate internucleotide linkages enabled nuclease-resistant DNA barcodes that allowed for in vivo sensing (Supplementary Fig. 1). crRNA–DNA barcode complementarity was critical for the collateral activity of *Lba*Cas12a in solution or injection in vivo (Figs. 2 and 5g, and Supplementary Figs. 11 and 12). Additionally, the molecular weight of DNA activators played an important role in vivo because they underwent characteristic single-exponential concentration decay after intravenous injection and size-dependent renal filtration from the blood (<10 kDa, peaking 1 h after delivery)^23–25^. Therefore, the ~7 kDa 20-mer DNA activator that effectively concentrated in urine outperformed longer PAM-containing sequences in *trans*-ssDNA cutting. In localized tumours, naturally occurring ctDNAs are often present at a low abundance and require extensive enrichment steps prior to sequencing. For CRISPR-based nucleic acid detection, inputs are often amplified by loop-mediated isothermal amplification (LAMP) or recombinase polymerase amplification (RPA) to improve the limit of detection^26,30^. At tolerable diagnostic doses (<0.3 mg per kg (body weight)), DNA barcodes liberated from the synthetic biomarker scaffold are readily accessible from compositionally simple, unprocessed urine without in vivo toxicity, based on normal histological staining and stable cytokine and chemokine levels upon sensor administration (Supplementary Fig. 13). The DNA detection sensitivity can be further improved by optimizing the ssDNA *trans*-cleavage activity of *Lba*Cas12a through enhanced base pairing between the DNA activator and modified crRNA.

The specialized substrate recognition and signal amplification of enzymes allows DNA-encoded SUBs to be fully modular, with both a designable input via a protease-responsive linker and a programmable output via CRISPR-Cas activity. We improved their on-target rate with functional biologics that can be extended to scaffolds with therapeutic efficacy, making it possible for combined therapy and diagnostics to monitor treatment responses in real time^51^. Besides proteases, expansion to additional enzymatic families will extend their utility to elucidate systemic activity-based networks^52^. In addition to systemic injection, synthetic biomarkers can be reformulated for oral, inhaled and topical delivery, and would allow for sensor administration without trained phlebotomists. Future PoC diagnostic systems may incorporate multiplexed sensing channels or surface-bound spots, and will benefit from computational tools to quantify more complex sensing results and improve diagnostic accuracy^53^. This way, patients could achieve the capacity to self-monitor disease progression to enable early detection and access to effective treatment. Through tailored applications, these programmable sensors may monitor other infectious and non-communicable diseases and guide treatment decisions to improve disease management in resource-limited settings.

## Methods

### Synthesis and characterization of peptides, oligonucleotides and peptide–oligonucleotide conjugates

All peptides and oligonucleotides were synthesized and HPLC purified by CPC Scientific and Integrated DNA Technologies (IDT), respectively. Peptide–oligonucleotide conjugates were generated by copper-free click chemistry. The conjugates were purified on an Agilent 1100 HPLC. Mass spectral analysis of the conjugates was performed on a Bruker model MicroFlex matrix-assisted laser desorption/ionization–time of flight spectrometry. Sequences of all molecules utilized are listed in Supplementary Tables 1 and 3.

### Cas12a fluorescent cleavage assay

*Lba*Cas12a (final concentration, 100 nM; New England Biolabs (NEB)) was incubated with 1× NEB Buffer 2.1, crRNA (250 nM; IDT) and complementary DNA activators (4 nM unless specifically described, in solution or spiked in urine; IDT) or urine samples collected from experimental animals, in a 50 μl reaction at 37 °C for 30 min. Reactions were diluted by a factor of 4 into 1× NEB Buffer 2.1 and ssDNA T_10_ F-Q reporter substrate (30 pmol; IDT) into a reaction volume of 60 μl per well and run in triplicate. *Lba*Cas12a activation was detected at 37 °C every 2 min for 3 h by measuring fluorescence with a Tecan Infinite Pro M200 plate reader (*λ*_ex_ = 485 nm, *λ*_em_ = 535 nm). Sequences of all oligonucleotides are listed in Supplementary Table 1. Fluorescence for background conditions (either no DNA activator input or no crRNA conditions) were used as negative controls. Cas12a ssDNase activity was calculated from the kinetics curve generated by the plate reader (fluorescence of the synthetic probe versus time). The initial reaction velocity (*V*_0_) corresponds to the slope of the kinetic curve’s linear phase (8–10 initial time points). Analysis was performed in Python v.3.9.

### Cas12a cleavage assay with lateral-flow readout

Samples were incubated for 30 min at 37 °C as in the Cas12a activation assay described above. Reactions were then diluted by a factor of 4 into 1× NEB Buffer 2.1 and ssDNA T_10_ FAM–biotin reporter substrate (1 pmol; IDT) into a reaction volume of 100 µl and incubated at 37 °C for 1–3 h. HybriDetect 1 lateral-flow strips were dipped into solution (20 μl of sample with 80 μl of Milenia Hybridetect buffer). The intensity of the bands was quantified in ImageJ v.1.49.

### Characterization of DNA activator concentration or length for Cas12a ssDNase activity

To identify the optimal length for detection with Cas12a, we tested truncated native and modified DNA activator lengths from 10 to 34 nt. To determine in vivo robustness, different lengths of phosphorothioate-modified DNA activators were injected at 1 nmol in BALB/c mice, and urine samples were collected 1 h after injection. Urine samples were used as DNA activators in the Cas12a fluorescent cleavage assay. Cas12a ssDNase activity triggered by each DNA activator was normalized to that of the 24-mer modified DNA activator.

### Fluidigm detection and data analysis

The Cas12 detection reactions were made into two separate mixes, assay mix and sample mix, for loading onto a microfluidic Gene Expression (GE) 96.96 integrated fluidic circuit (IFC) (Fluidigm): the assay mix contained 10 μM *Lba*Cas12a (NEB), 1× Assay Loading Reagent (Fluidigm), 1× NEB Buffer 2.1 and 1 μM crRNA for a total volume of 16 μl per reaction. The sample mix contained 25.2 U RNase inhibitor (NEB), 1× NEBuffer 2.1, 1× ROX Reference Dye (Invitrogen), 1× GE Sample Loading Reagent (Fluidigm), 9 mM MgCl_2_ and 500 nM quenched synthetic fluorescent DNA reporter (FAM–T_10_–3IABkFQ, IDT) for a total volume of 12.6 μl. Syringe and 4 μl of assay or sample mixtures were then loaded into their respective locations on a microfluidic GE 96.96 IFC and were run according to the manufacturer’s instructions. The IFC was loaded onto the Juno (Fluidigm) where the ‘Load Mix’ script was run. After proper IFC loading, images were collected over a 3 h period using a custom protocol on Biomark HD.

To analyse the data generated by the Fluidigm system, we plotted reference-normalized background-subtracted fluorescence for guide–target pairs. For a guide–target pair (at a given time point, *t*, and target concentration), we first computed the reference-normalized value as (median (*P*_t_ − *P*_0_)/(*R*_t_ − *R*_0_)) where *P*_t_ is the guide signal (FAM) at the time point, *P*_0_ is its background measurement before the reaction, *R*_t_ is the reference signal (ROX) at the time point, *R*_0_ is its background measurement, and the median is taken across replicates. Data was visualized using Python 3, R 4 and Prism 9.

### Cloning and expression of recombinant nanobodies

Double-stranded gBlocks gene fragments encoding the nanobody of interest with flanking NcoI and BlpI restriction sites were ordered from IDT. The gene fragments were cloned into Novogen pET-28a(+) expression vector at NcoI and BlpI restriction sites and transformed into SHuffle T7 competent *Escerichia coli* (NEB). Bacterial colonies encoding the correct gene inserts were confirmed with Sanger sequencing. For subsequent recombinant protein production, a 500 ml secondary culture of SHuffle T7 competent *E. coli*. encoding the nanobody gene of interest was grown in kanamycin-supplemented LB broth at 37 °C from an overnight 3 ml primary culture until the optical density at 600 nm (OD600) reached about 0.6–0.8. Nanobody expression was then induced with an addition of isopropyl β-d-1-thiogalactopyranoside (IPTG) (0.4 mM final concentration). The culture was incubated at 27 °C for 24 h. The bacterial pellet was lysed with B-PER complete bacteria protein extraction reagent (Thermo Fisher Scientific), then purified via standard immobilized metal affinity chromatography (IMAC) with Ni-NTA agarose (Qiagen). The nanobody product was confirmed via SDS–polyacrylamide gel electrophoresis analysis. Sequences of nanobodies utilized in this study are listed in Supplementary Table 3.

### Synthesis of DNA-encoded synthetic urine biomarker with a nanobody core

Nanobody (2 mg) was incubated at room temperature overnight in Pierce immobilized TCEP disulfide reducing gel (7.5% v/v) (Thermo Fisher Scientific) to selectively reduce C-terminal cysteine following a previously established protocol^37^. The reduced C-terminal cysteine (1 equiv.) was reacted with sulfo DBCO-maleimide crosslinker (4 equiv.) (Click Chemistry Tools) in PBS (pH 6.5, 1 mM EDTA) at room temperature for 6 h after which the excess crosslinker was removed with a disposable PD-10 desalting column (GE Healthcare Bio-Sciences). DBCO-functionalized nanobody was further refined by ÄKTA fast-protein liquid chromatography (GE Healthcare). DNA reporter conjugation was performed by incubating DBCO-functionalized nanobody (1 equiv.) with azide-functionalized DNA reporter (1.1 equiv.) in PBS (pH 7.4) at room temperature for 24 h. Excess DNA reporter was removed via size exclusion chromatography. The product was confirmed via SDS–polyacrylamide gel electrophoresis analysis and quantified with the Quant-iT OliGreen ssDNA Assay Kit. Sequences of DNA-barcoded synthetic urine biomarkers utilized in this study are listed in Supplementary Table 4.

### Synthesis of DNA-encoded synthetic urine biomarkers with polymeric cores

Multivalent PEG (40 kDa, eight-arm) containing maleimide-reactive handles (JenKem Technology) was dissolved in 100 mM phosphate buffer (pH 7.0) and filtered (pore size, 0.2 μm). After filtration, the cysteine-terminated peptide–DNA conjugates were added at 2-fold molar excess to the PEG and reacted for at least 4 h at room temperature. Unconjugated molecules were separated using size-exclusion chromatography with a Superdex 200 Increase 10/300 GL column on an ÄKTA fast protein liquid chromatograph (GE Healthcare). The purified nanosensors were concentrated by spin filters (molecular weight cut-off, 10 kDa; Millipore), and quantified with a Quant-iT OliGreen ssDNA Assay Kit (Thermo Fisher Scientific). Fluorescence was read on a Tecan Infinite Pro M200 Quant-iT plate reader at *λ*_ex_ = 485 nm, *λ*_em_ = 535 nm. Particles were stored at 4 °C in PBS. Dynamic light scattering (Zeta Sizer Nanoseries, Malvern Instruments) was used to characterize the hydrodynamic diameter of nanoparticles. Sequences of DNA-barcoded synthetic urine biomarkers are listed in Supplementary Table 4.

### Cryogenic transmission electron microscopy

The 5-plex polymeric core-based DNA-SUBs were pooled and concentrated to 0.5 mg ml^−1^ by DNA concentration. Samples were loaded on a lacey copper grid coated with a continuous carbon film. The grid was then mounted on a Gatan 626 single-tilt cryoholder which was placed in the transmission electron microscope column. The samples were cooled down by liquid nitrogen and kept cold during transfer into the microscope (JEOL 2100 FEG microscope set at 200 kV; magnification, 10,000–60,000). All images were recorded using a Gatan 2kx2k UltraScan charge-coupled device camera.

### Transcriptomic and proteomic analysis

RNA-Seq data of human colon adenocarcinoma were generated by the Cancer Genome Atlas Research Network (http://cancergenome.nih.gov). Differential expression analyses were carried out with DESeq2 1.10.1. Proteomic data on the composition of extracellular matrix in human colon cancers and normal colon tissues were obtained by mass spectrometry analysis of extracellular matrix components and are available from Matrisome (http://matrisomeproject.mit.edu/).

### Cell culture

The mouse cell line MC26-LucF (carrying firefly luciferase, from Kenneth K. Tanabe Laboratory, Massachusetts General Hospital) was cultured in DMEM (Gibco) medium supplemented with 10% (v/v) fetal bovine serum (Gibco), 1% (v/v) penicillin/streptomycin (CellGro) at 37 °C and in 5% CO_2_. Human cell lines PC-3 (ATCC CRL-1435) were grown in RPMI1640 (Gibco) supplemented with 10% (v/v) fetal bovine serum and 1% (v/v) penicillin/streptomycin. RWPE1 (ATCC CRL-11609) cells were cultured in keratinocyte serum-free medium (Gibco) supplemented with 2.5 µg human recombinant epidermal growth factor and 25 mg bovine pituitary extract. All cell lines tested negative for mycoplasma contamination.

### Animal models

All animal studies were approved by the Massachusetts Institute of Technology (MIT) committee on animal care (MIT protocols 0420-023-23 and 0220-010-23). All experiments were conducted in compliance with institutional and national guidelines and supervised by the Division of Comparative Medicine (DCM) of MIT staff.

Female BALB/c (BALB/cAnNTac, 6–8 weeks of age; Taconic Biosciences), female NCr nude (CrTac:NCr-Foxn1^nu^, 4–5 weeks of age, Taconic Biosciences), and female and male *Kras*^LSL−G12D/+^;*Trp*53^fl/fl^ C57L/B6 (KP) mice (8–16 weeks of age; gift from Tyler Jacks Laboratory, MIT) were used for experiments. Mice were maintained in the Koch Cancer Institute animal facility, with a 12 h light/12 h dark cycle (07:00–19:00), at ~18–23 °C and ~50% humidity. Autoclaved water and standard chow diet were accessible at all times. NCr nude mice were housed in immunodeficient-only rooms, in autoclaved cages and paper bedding and handled with sterile techniques. The health status of mice was checked weeklym and daily when tumours were >5 mm in diameter. Mice were euthanized according to the veterinarians’ criteria (for example, tumour size >1 cm, poor body conditions). We used a sample size of at least three mice per group. Group size is greater than or equal to five when comparing urinary barcode levels (two-sided *t*-test, α set at 0.05). Littermates of the same sex were randomly assigned to experimental and control groups.

To establish the CRC lung tumours, BALB/c female mice were inoculated by intravenous injection with the luciferase-expressing MC26-Fluc cell line (100,000 cells per mouse). Tumour progression was monitored weekly using an IVIS imaging system (PerkinElmer) and quantified on Living Image (PerkinElmer). To establish the PCa xenograft model, NCr nude female mice were inoculated with human PC-3 cell lines (5 million cells per flank, 2 flanks per mouse) while under isoflurane anaesthesia. Cells were prepared in 30% Corning Matrigel Membrane Matrix (Thermo Fisher Scientific) and low-serum media (Opti-MEM, Gibco). Tumours were measured weekly and experiments were conducted once flank tumours reached approximately 5 mm in length or width (~200 mm^3^) or 3 weeks after inoculation. Tumour volume was calculated by caliper measurement of the length and width of the flank; volume calculation followed the equation *f*_*x*_ = (width^2^ × length)/2, where length is the longer segment.

To induce autochthonous lung tumours, we first generated *Kras*^LSL−G12D/+^;*Trp*53^fl/fl^ C57L/B6 (KP) mice^42,43^ in which the activation of an oncogenic allele of *KRAS* is sufficient to initiate the tumorigenesis process, and additional deletion or point mutation of *Trp53* substantially enhances tumour progression, leading to a more rapid development of adenocarcinomas that have features of a more advanced disease. Lung tumours were initiated by intratracheal administration of 50 μl adenovirus-SPC-Cre (2.5 × 10^8^ plaque-forming units in Opti-MEM with 10 mM calcium chloride in female or male KP mice aged between 8 and 16 weeks) under isoflurane anaesthesia. Control cohorts consisted of age- and sex-matched mice that also underwent intratracheal administration of AdenoCre. The KP mice were maintained without further intervention to allow for tumour growth until experiments were performed.

### Analysis of urinary DNA-barcode-activated Cas12a cleavage assay

ssDNAs (1 nmol), 5-plex DNA-barcoded PEG sensors (0.2 nmol each, 1 nmol by DNA barcode concentration in total) or DNA-barcoded nanobody sensors (1 nmol by DNA barcode concentration) were intravenously injected into experimental mice. Urine samples (100–200 μl per mouse) were collected at 12:00 each day, 1 h after DNA or sensor injection, and were assayed for Cas12a activation to determine the initial reaction velocity (*V*_0_). Mean normalization was performed on *V*_0_ values to account for animal-to-animal variation in urine concentration. In the Cas12a cleavage assay that utilized a fluorescent reporter, the *y* axis represents mean normalized *V*_0_tumour-bearing animals_/mean normalized *V*_0_control animals_. The same urine sample was then utilized to perform the Cas12a cleavage assay with LFA readout. The resulting paper strips were aligned and scanned simultaneously. Band intensity was quantified with ImageJ v.1.49v.

### Biodistribution and pharmacokinetics studies

Near-infrared-dye-labelled agents were used to minimize interference from autofluorescent background in vivo. BALB/c mice were intravenously injected with Cy5-labelled modified or native DNA molecules (1 nmol) and urine samples were collected at 30 min and 1, 2, 3, 4 h after injection. Nanobody–DNA conjugates were coupled with sulfo-Cyanine7 NHS ester (Lumiprobe, 2 dye equiv. of protein), reacted overnight, purified by spin filtration and injected intravenously into PC-3 tumour-bearing nude mice. After 24 h, mice were euthanized and necropsy was performed to remove the tumours, lungs, heart, kidneys, liver and spleen. Urine, blood and organs were scanned using IVIS and Odyssey CLx imaging systems (LI-COR). Organ fluorescence was quantified by ImageStudio in the Odyssey CLx. Blood circulatory kinetics were monitored in BALB/c mice by serial blood draws at 10 min, 30 min, 2 h and 3 h after intravenous injection of Cy5-labelled DNA or PEG at 1 nmol dye per mouse. Blood for pharmacokinetics measurements was collected using tail vain bleeds. Blood was diluted in PBS with 5 mM EDTA to prevent clotting, centrifuged for 5 min at 5,000*g*, and fluorescent reporter concentration was quantified in 384-well plates relative to standards on the Odyssey CLx.

### Histology, immunohistochemistry and immunofluorescence studies

Paraffin-embedded tissues were preserved in 4% paraformaldehyde overnight and stored in 70% ethanol prior to embedding into paraffin. Snap-frozen tissues were preserved in 2% paraformaldehyde for 2 h, stored in 30% sucrose overnight and frozen in optimum cutting temperature (OCT) compound at −80 °C. Snap-frozen lungs were processed through intratracheal injection of 50:50 OCT in PBS immediately after the animal had been euthanized by isoflurane overdose. The lungs were slowly frozen with OCT embedding in an isopentane/liquid nitrogen bath. Samples were sectioned into 6 µm slices. For immunohistochemistry studies, slides were stained with primary antibodies in accordance with the manufacturer’s instructions, followed by a Rabbit-on-Rodent HRP-Polymer used as received (Biocare Medical). For immunofluorescence studies, after blocking with 5% goat serum, 2% BSA and 0.1% Triton X-100 in PBS for 1 h, sections were stained with a primary antibody in 1% BSA in PBS overnight at 4 °C. Alexa Fluor conjugated secondary antibodies were incubated at 1 μg ml^−1^ in 1% BSA in PBS for 30 min at room temperature. Slides were sealed with ProLong Antifade Mountant (Thermo Fisher Scientific), and digitized and analysed using a 3D Histech P250 high-capacity slide scanner (PerkinElmer). Histological toxicity was evaluated by a veterinary pathologist who was blinded to the treatment groups. Primary antibodies and dilutions used are listed in Supplementary Table 5.

### RNA extraction and real-time quantitative polymerase chain reaction

PC-3 and RWPE1 cells were cultured and collected after trypsinization. Tissue samples were collected by necropsy after mice had been euthanized and were immediately kept in RNAlater RNA Stabilization Reagent (Qiagen). RNA from cell pellets or cryoground tissue samples was extracted using an RNeasy Mini Kit (Qiagen). RNA was reverse transcribed into cDNA using Bio-Rad iScript Reverse Transcription Supermix on a Bio-Rad iCycler. Quantitative polymerase chain reaction amplification of the cDNA was measured after mixing with Taqman gene expression probes and Applied Biosystems TaqMan Fast Advanced Master Mix (Thermo Fisher Scientific) according to the manufacturer’s instructions. Quantitative polymerase chain reaction was performed on a Bio-Rad CFX96 Real Time System C1000 Thermal Cycler.

### Recombinant protease substrate and tissue lysate proteolytic cleavage assays

Fluorogenic protease substrates with fluorescence (FAM) and quencher (CPQ2) were synthesized by CPC Scientific. Recombinant proteases were purchased from Enzo Life Sciences and R&D Systems. Tissue samples were homogenized in PBS and centrifuged at 4 °C for 5 min at 6,000*g*. Supernatant was further centrifuged at 14,000*g* for 25 min at 4 °C. Protein concentration was measured using a Thermo Fisher BCA Protein Assay Kit and prepared at 2 mg ml^−1^. Assays were performed in 384-well plates in triplicate in enzyme-specific buffer with peptides (1 µM) and proteases (40 nM for recombinant protease assay)/cell lysates (0.33 mg ml^−1^ for tissue lysate assay) in 30 µl at 37 °C. Fluorescence was measured at *λ*_ex_ = 485 nm, *λ*_em_ = 535 nm on a Tecan Infinite 200pro microplate reader. Enzymes and buffer conditions are listed in Supplementary Table 6.

### PSA enzyme-linked immunosorbent assay

Approximately 200 μl of blood was collected from the lateral saphenous vein of experimental animals and blood cells were pelleted immediately by centrifugation at 14,000*g* for 25 min at 4 °C. Plasma was stored at −80 °C prior to PSA quantification. PSA levels were measured using the PSA Quantikine ELISA kit according to the manufacturer’s protocols (R&D Systems).

### Statistical analysis and reproducibility

Statistical analyses were conducted in GraphPad Prism 9. Data were displayed as means with s.e.m. Differences between groups were assessed using parametric and non-parametric group comparisons when appropriate with adjustment for multiple-hypothesis testing. Specifically, data groups were first tested for normality by the Kolmogorov–Smirnov normality test with the Dallal–Wilkinson–Lillie test for *P* value. Results were then tested for statistical significance by unpaired two-tailed *t*-test (parametric) for two-group comparisons and analysis of variance for multiple-group comparisons. Non-parametric analyses were conducted by unpaired two-tailed Mann–Whitney test. Sample sizes and statistical tests are specified in the figure legends. All experiments were repeated independently at least twice with similar results. Note that experiments were repeated and visualized by two independent researchers when results from representative experiments (such as histological or fluorescent micrographs) are shown.

### Reporting summary

Further information on research design is available in the Nature Portfolio Reporting Summary linked to this article.

## Online content

Any methods, additional references, Nature Portfolio reporting summaries, source data, extended data, supplementary information, acknowledgements, peer review information; details of author contributions and competing interests; and statements of data and code availability are available at 10.1038/s41565-023-01372-9.

## Supplementary information

Supplementary Information Supplementary Figs. 1–13, Tables 1–6 and code.

Reporting Summary

## Data Availability

The Cancer Genome Atlas (http://cancergenome.nih.gov) and Matrisome (http://matrisomeproject.mit.edu/) are open access resources. The datasets and codes generated and analysed during the current study are available in the Zenodo repository (https://zenodo.org/deposit/7686811). All data that support the findings of this study are available within the Article and its Supplementary Information or from the corresponding author upon reasonable request. Source data are provided with this paper.

## Source data

Source Data Fig. 2 Statistical Source Data
Source Data Fig. 3 Statistical Source Data
Source Data Fig. 4 Statistical Source Data
Source Data Fig. 5 Statistical Source Data

## Code availability

Python v.3.9 package for enzyme kinetics analysis is available through GitHub: https://github.com/nharzallah/NNanotech-Kinetic. Custom analysis codes for Fluidigm microfluidics are on GitHub: https://github.com/broadinstitute/mcarmen.
