# Supplementary material for: CRISPR-Cas-amplified urinary biomarkers for multiplexed and portable cancer diagnostics
Source: Nat Nanotechnol. 2023 Apr 24;18(7):798–807. doi: 10.1038/s41565-023-01372-9 (PMC10359190; doi:10.1038/s41565-023-01372-9)
Supplement: Supplementary file 1 — Supplementary Figs. 1–13, Tables 1–6 and code. [file 41565_2023_1372_MOESM1_ESM.pdf]

nature nanotechnology

Article https://doi.org/10.1038/s41565-023-01372-9

CRISPR-Cas-amplified urinary biomarkers for multiplexed and portable cancer diagnostics

In the format provided by the authors and unedited

Table of Content

Supplementary Figure 1. Collateral activity of LbaCas12a activated by different types of DNA activators. ···· 2

Supplementary Figure 2. Dose-dependent LbaCas12a activation by chemically stabilized DNA activators. ···· 4

Supplementary Figure 3. Dose-dependent LbaCas12a activation in paper-based lateral flow assays. ···· 6

Supplementary Figure 4. Characterizations of DNA-conjugated nanobody in vitro and in vivo. ···· 7

Supplementary Figure 5. Characterizations of DNA-encoded synthetic urinary biomarker with a polymeric PEG core. ···· 8

Supplementary Figure 6. Histology of major organs of CRC lung metastasis model. ···· 9

Supplementary Figure 7. Identification of dysregulated proteases in CRC to select peptide substrates for in vivo sensors. ···· 10

Supplementary Figure 8. Differential cleavage of selected peptide substrates when treated with tumor or normal tissues. ···· 11

Supplementary Figure 9. DNA-encoded synthetic urinary biomarkers for multiplexed disease monitoring. ···· 12

Supplementary Figure 10. Specificity of multiplexed DNA-encoded synthetic urinary biomarkers tested in PC-3 derived prostate cancer. ···· 13

Supplementary Figure 11. Massively in-parallel CRISPR-Cas-mediated DNA detection on the Fluidigm microfluidics platform. ···· 15

Supplementary Figure 12. Massively in-parallel CRISPR-Cas-mediated DNA detection in solution on the Fluidigm microfluidics platform. ···· 16

Supplementary Figure 13. In vivo toxicity of DNA-encoded synthetic urinary biomarkers. ···· 17

Supplementary Table 1. Nucleic acid sequences used in this study. ···· 18

Supplementary Table 2. Activation of Cas12a with native and modified DNA oligos in vitro and in vivo. ···· 23

Supplementary Table 3. Peptide and protein sequences used in this study. ···· 24

Supplementary Table 4. Sequences of DNA-barcoded synthetic urinary biomarkers. ···· 25

Supplementary Table 5. List of all primary antibodies. ···· 26

Supplementary Table 6. List of buffers for proteolytic cleavage assays. ···· 27

Supplementary Code. Enzymatic kinetics analysis. ···· 28

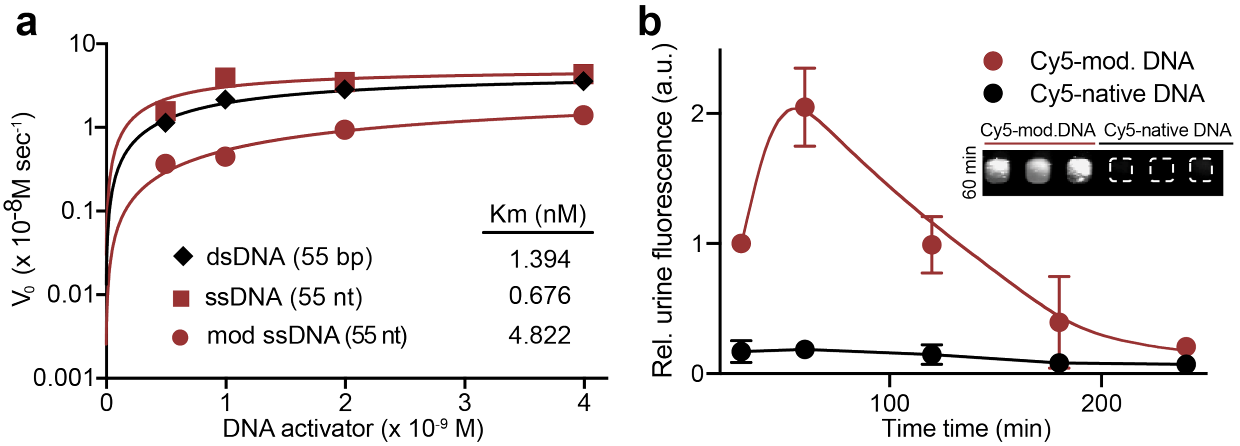

Supplementary Figure 1. Collateral activity of *Lba*Cas12a activated by different types of DNA activators.

(**a**) *Trans*-cleavage rates of Cas12a upon activation of different type of DNA activator-crRNA pairs were determined in the Cas12a fluorescent cleavage assay. Representative Michaelis-Menten plot of *Lba*Cas12a-catalyzed ssDNA *trans*-cleavage using a native dsDNA, ssDNA, or fully phosphorothioate-modified ssDNA activator. The initial reaction velocity (V_0) is determined from the slope of the curve at the beginning of a reaction. (**b**) The urine signal after systemic administration of modified and native 20-mer DNAs showing amplification kinetics of modified DNA that surpassed the steady-state concentration of its native DNA counterpart. Signal maximized at 1 h after DNA administration. Image shows urine samples on a 384-well plate visualized on the LI-COR Odyssey CLx system. Urine fluorescence was normalized to that of the first timepoint (30 min after DNA injection) of Cy5-modified DNA injected BALB/c mice (n=3 animals per condition, data are shown as mean ± s.e.m.).

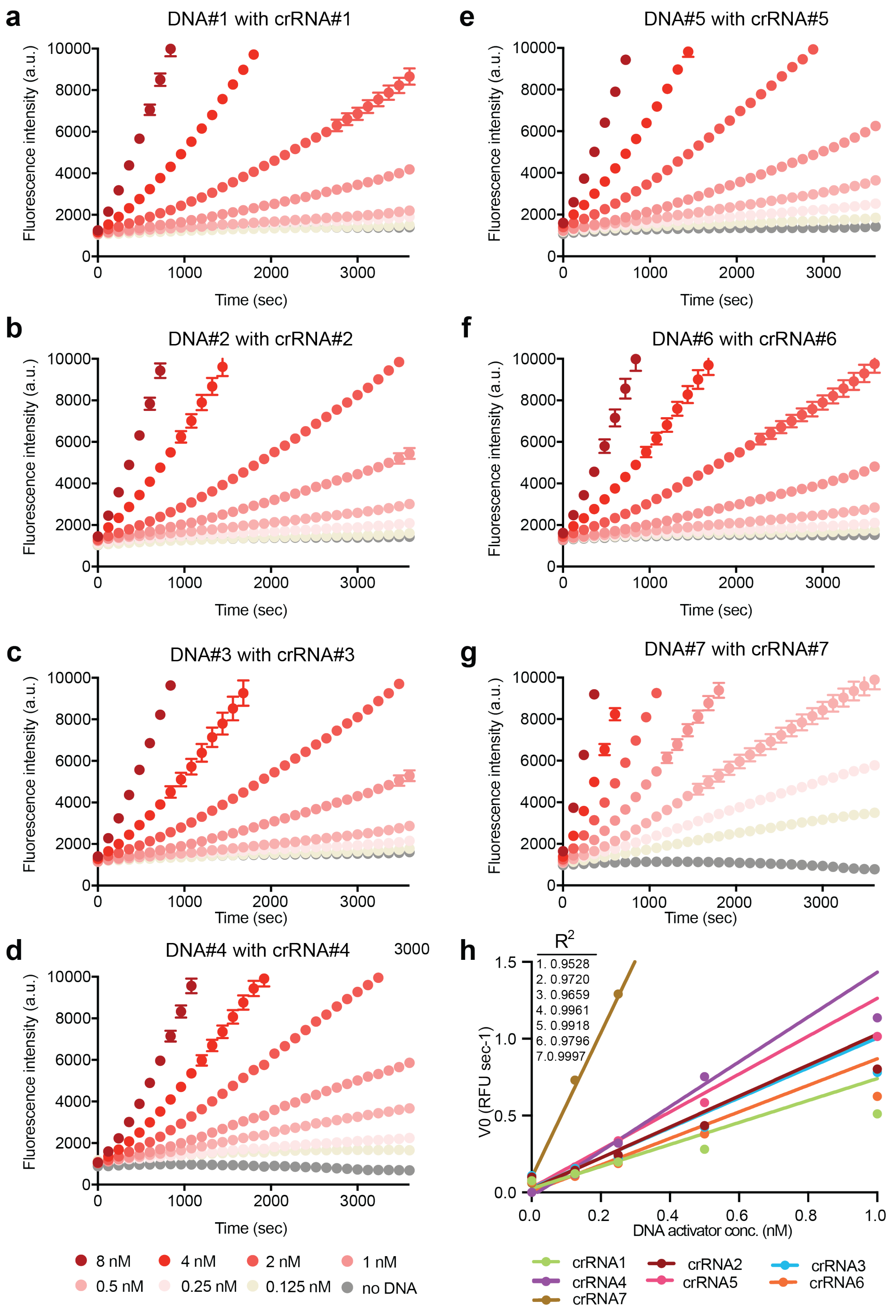
a
DNA#1 with crRNA#1
b
DNA#2 with crRNA#2
c
DNA#3 with crRNA#3
d
DNA#4 with crRNA#4
3000
e
DNA#5 with crRNA#5
f
DNA#6 with crRNA#6
g
DNA#7 with crRNA#7
Fluorescence intensity (a.u.)
Time (sec)
0
2000
4000
6000
8000
10000
1000
3000
8 nM
4 nM
2 nM
1 nM
0.5 nM
0.25 nM
0.125 nM
no DNA
h
R^2
1. 0.9528
2. 0.9720
3. 0.9659
4. 0.9961
5. 0.9918
6. 0.9796
7.0.9997
V0 (RFU sec-1)
DNA activator conc. (nM)
0.0
0.2
0.4
0.5
0.6
0.8
1.0
1.5
crRNA1
crRNA2
crRNA3
crRNA4
crRNA5
crRNA6
crRNA7

Supplementary Figure 2. Dose-dependent *Lba*Cas12a activation by chemically stabilized DNA activators.

(**a-g**) *Lba*Cas12a catalyzed ssDNA *trans*-cleavage after activation by phosphorothioate-modified ssDNA activators. *Trans*-cleavage rates of Cas12a upon activation of different modified ssDNA activator-crRNA pairs were determined in the Cas12a fluorescent cleavage assay. Assays were performed with different concentration of modified ssDNA activator (8 nM, 4 nM, 2 nM, 1 nM, 0.5 nM, 0.25 nM, 0.125 nM, and 0 nM; n=3 independent samples, data are shown as mean ± s.e.m.). (**h**) The initial reaction velocity (V_0) is determined from the slope of the curve at the beginning of a reaction in (**a-g**) and plotted to determine the linear range of assay performance. Linear regions were shown in V_0 of reactions for all modified ssDNA activator-crRNA pairs within 1 nM of DNA activators. DNA activator 1, 2, 3, 5, 6 were selected for construction of *in vivo* sensors because of their similarity in assay performance. Sequences of oligonucleotides were shown in Supplementary Table 1.

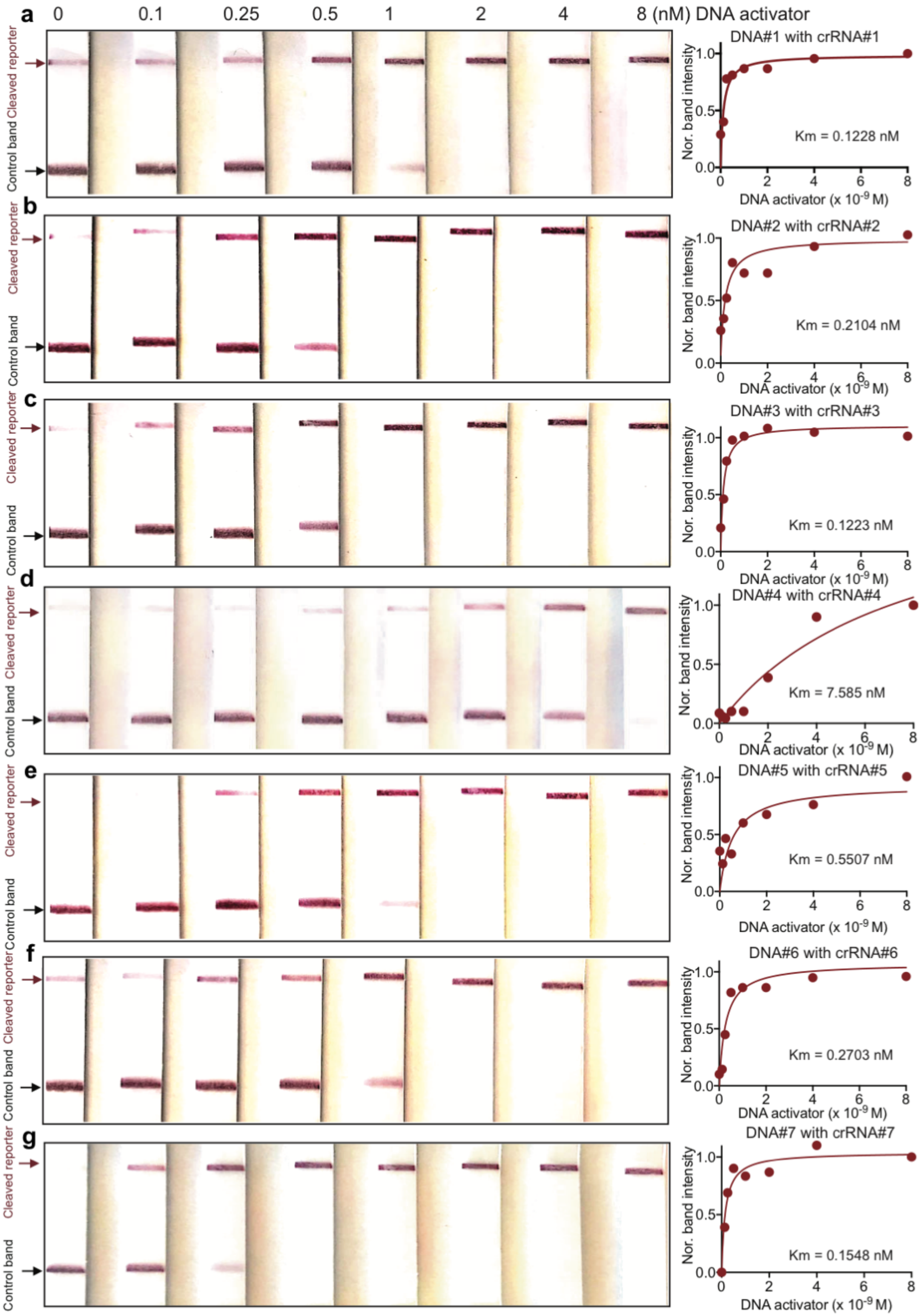

a
0
0.1
0.25
0.5
1
2
4
8 (nM) DNA activator
Cleaved reporter
Control band
DNA#1 with crRNA#1
Km = 0.1228 nM
b
DNA#2 with crRNA#2
Km = 0.2104 nM
c
DNA#3 with crRNA#3
Km = 0.1223 nM
d
DNA#4 with crRNA#4
Km = 7.585 nM
e
DNA#5 with crRNA#5
Km = 0.5507 nM
f
DNA#6 with crRNA#6
Km = 0.2703 nM
g
DNA#7 with crRNA#7
Km = 0.1548 nM
Nor. band intensity
DNA activator (x 10^-9 M)

Supplementary Figure 3. Dose-dependent *Lba*Cas12a activation in paper-based lateral flow assays.

(**a-g**) *Trans*-cleavage rates of Cas12a upon activation of different modified ssDNA activator-crRNA pairs were determined in the Cas12a lateral flow assay. Assays were performed with different concentrations of modified ssDNA activator (8 nM, 4 nM, 2 nM, 1 nM, 0.5 nM, 0.25 nM, 0.1 nM and 0 nM) and dual-labeled FAM-T_{10}-Biotin reporter. Resulting solution was mixed with HybriDetect 1 assay buffer. HybriDetect 1 lateral flow strips were dipped into solution and intensity of cleaved reporter bands was quantified in ImageJ and plotted to fit Michaelis-Menten kinetics. Consistent with the Cas12a fluorescent cleavage assays shown in Supplementary Fig. 2, linear regions were found within 1 nM of DNA activators for all modified ssDNA activator-crRNA pairs tested. Sequences of oligonucleotides were shown in Supplementary Table 1 (Mod DNAs and crRNAs 1-7).

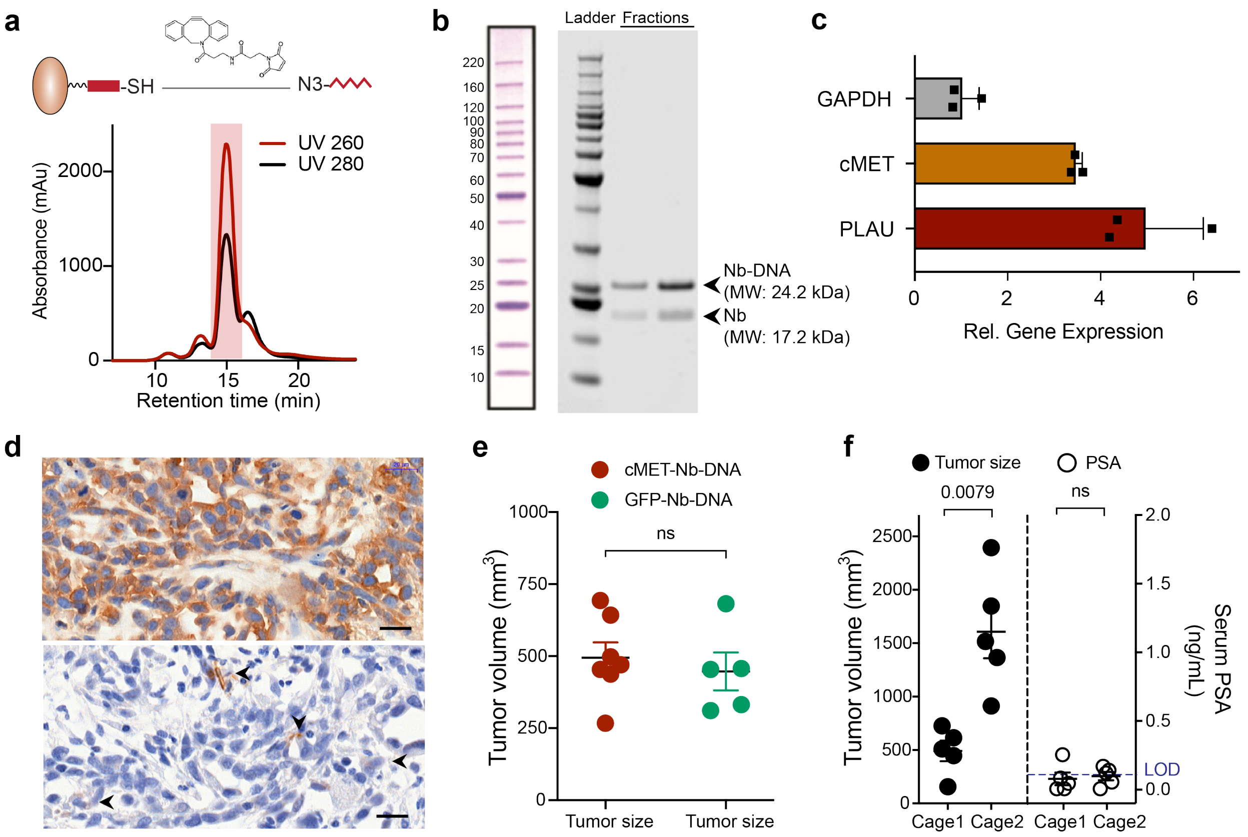

Supplementary Figure 4. Characterizations of DNA-conjugated nanobody *in vitro* and *in vivo*.

(**a**) Separation of DNA-conjugated cMET nanobody in a size exclusion chromatography. UV 260 nm, the elution curve of oligonucleotides; UV 280 nm, the elution curve of proteins. Red shading indicates the elution of the DNA-nanobody conjugate that has absorbance at both 260 nm and 280 nm. (**b**) SDS-PAGE analysis of the DNA-nanobody conjugate showing predicted molecular weight. (**c**) Relative expression of cMET, the biomarker that the nanobody targets, and PLAU, the protease triggering the DNA barcode release, in prostate cancer line PC-3 compared with normal prostate epithelial line RWPE1 (n=3 biologically independent cells, data are shown as mean ± s.e.m.). (**d**) Immunohistochemical staining of cMET and PLAU in PC-3 flank tumors. Brown, positive staining. Blue, nuclei. Scale bar = 200 μm. (**e**) Caliper quantification of tumor sizes of animals shown in Fig. 3d and 3e. Tumor-bearing mice were injected with different types of DNA-conjugated nanobodies (Data are shown as mean ± s.e.m.; n=5 mice per group treated with GFP-Nb-DNA, n=7 mice per group treated with cMET-Nb-DNA; two-tailed unpaired *t*-test with Welch's correction, n.s.= not significant, *P*= 0.5882). (**f**) Subcutaneously implanted PC-3 tumors were sized (left half of the graph) and serum PSA collected from these tumor-bearing mice were measured by ELISA (right half of the graph). Average serum PSA concentration did not exceed the limit of detection (LOD) even till the total tumor volumes of two flanks of PC-3 tumors reached over 1,000 mm^3 in one animal. Data are shown as mean ± s.e.m, n=5 mice per group, two-tailed unpaired *t*-test with Welch's correction. n.s.= not significant, *P*=0.7356.

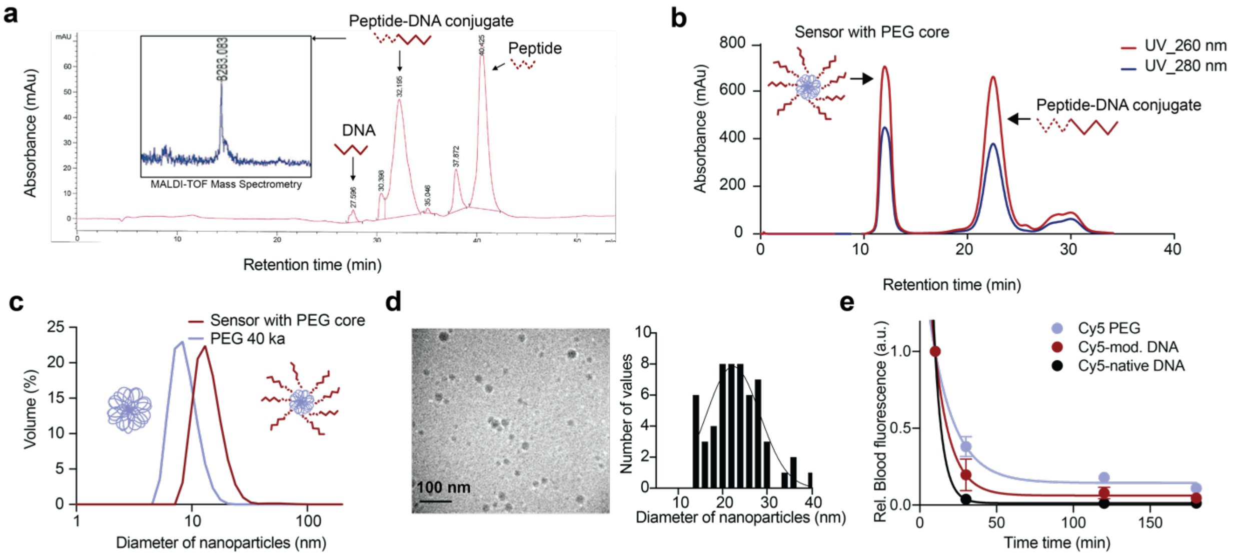

Supplementary Figure 5. Characterizations of DNA-encoded synthetic urinary biomarker with a polymeric PEG core.

(**a**) Characterization of the representative DNA-PAP7-SUB with a PEG core. HPLC purification of peptide-DNA (PAP7-DNA2) conjugate. The conjugate was analyzed in mass-spectrometry and showed expected molecular weight (8,283 Da). (**b**) FPLC purification of sensor showed separation of functionalized sensor and unbounded peptide-DNA conjugate. (**c**) Dynamic light scattering analysis showed the increase of particle size when PEG core (8.3 ± 2.5 nm) was functionalized as DNA-SUB sensors (13.1 ± 4 nm). (**d**) Cryogenic transmission electron microscopy (cyro-TEM) image of the 5-plex DNA-SUB cocktail (0.5 mg/mL by DNA concentration) for particle sizing. The histogram shows particle size distribution (n=60 particles). The average diameter of the sensor cocktail was 23.5 ± 6.4 nm. The solid line represents the Gaussian fit of the size distribution. (**e**) Plasma half-life shows rapid clearance of native DNA molecules and prolonged half-life of the modified DNA and PEG scaffold in healthy BALB/c mice (n=3 animals per group, data are presented as mean ± s.e.m.).

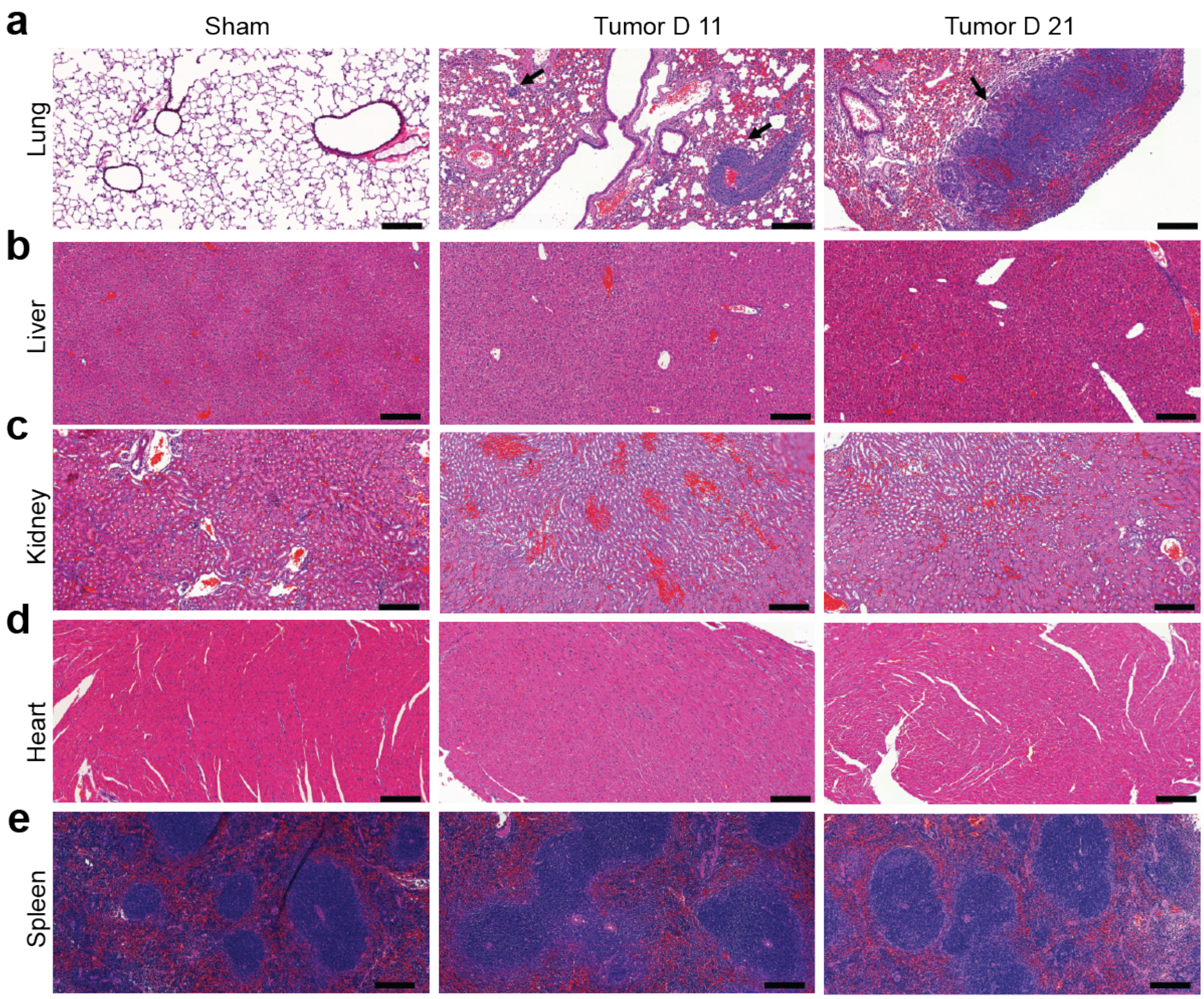

Supplementary Figure 6. Histology of major organs of CRC lung metastasis model. Immunocompetent BALB/c mice were injected with MC26-Fluc cells (tumor) or saline (sham) intravenously. (**a-e**) Organs (lung, liver, kidney, heart and spleen) were collected at 11 and 21 days after administration. Organs were fixed, embedded in paraffin, and stained with hematoxylin and eosin. Study was done with n=3 mice per time point and images from a representative animal are shown. Scale bar = 100 μm. Arrows indicate tumor nodules in the lung.

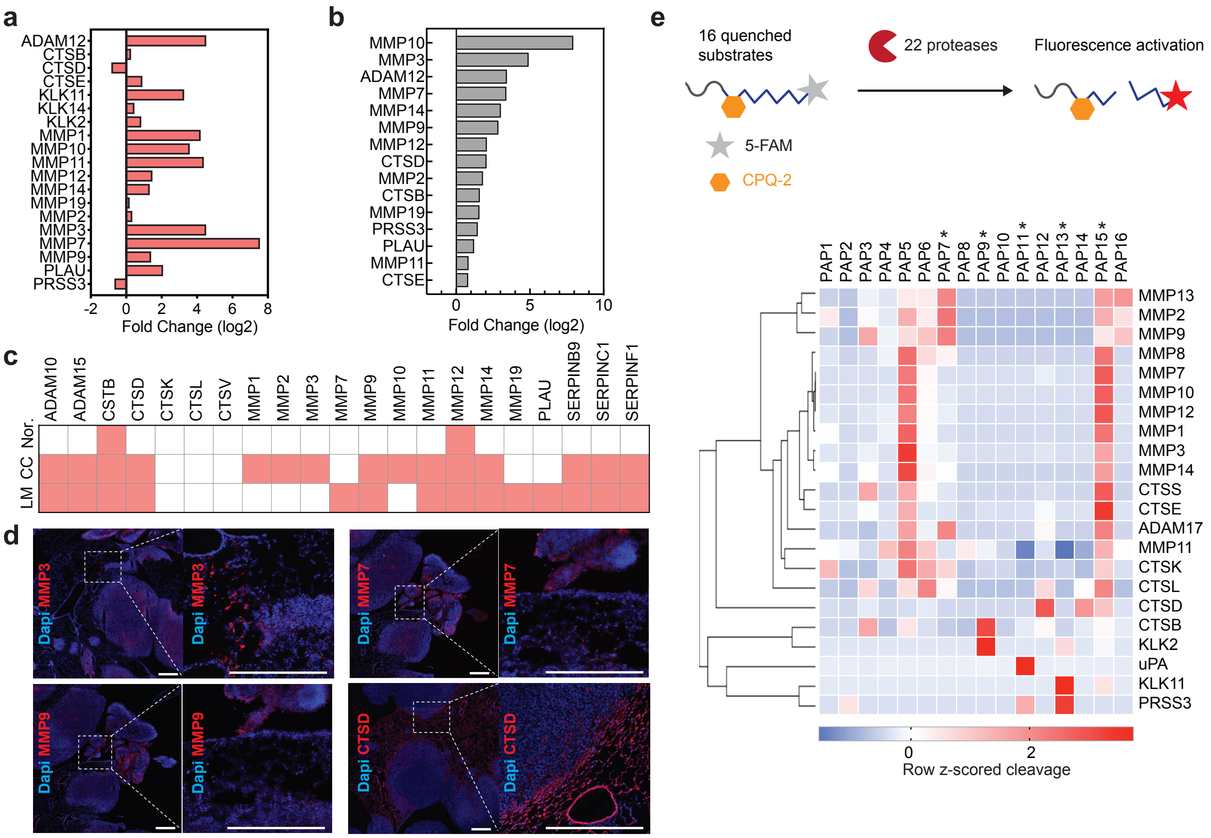

Supplementary Figure 7. Identification of dysregulated proteases in CRC to select peptide substrates for *in vivo* sensors.

(**a**) Analysis of differentially expressed proteases in CRC samples and normal adjacent tissues. Data available from the Cancer Genome Atlas. (**b**) RT-qPCR validation of proteases in the tumor-bearing lung from BALB/c mice injected with MC26-Fluc cells in comparison of normal lung from BALB/c mice injected with saline. (**c**) Typical proteases identified in the matrix of primary human colon cancer (CC) and their liver metastases (LM), in comparison to normal colon (Nor.) tissue. Pink, presence; white, absence. Data available from the Matrisome project (http://matrisomeproject.mit.edu/). (**d**) Immunofluorescence staining of proteases in the tumor bearing lung tissue sections. Staining of MMP3, MMP7, MMP9 and Cathepsin D (CTSD) is shown in red. Nuclei are counterstained blue with DAPI. Scale bar = 100 µm. (**e**) 16 FRET-paired protease substrates, each consisting of a peptide sequence flanked by a FAM fluorophore and a CPQ-2 quencher, were screened against 22 recombinant proteolytic enzymes. Details of each enzyme can be found in Supplementary Table 6. Lower, FRET signal was monitored by kinetic plate reader and the z-scored cleavage rate were subjected to heatmap and Hierarchical Clustering on Morpheus (https://software.broadinstitute.org/morpheus). Asterisk, peptide substrates selected for construction of *in vivo* sensors because of their broad coverage of metallo-, serine and aspartic protease activities.

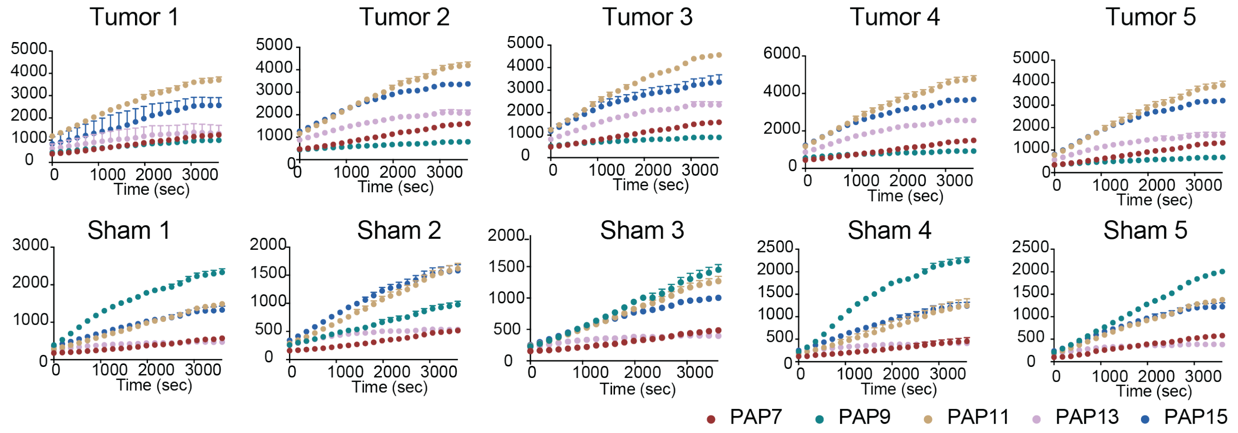

Supplementary Figure 8. Differential cleavage of selected peptide substrates when treated with tumor or normal tissues.

Selected FRET-paired protease substrates (PAP 7, PAP 9, PAP 11, PAP 13 and PAP 15) were incubated against tissue lysates from tumor bearing lung (tumor, upper) or normal lung (sham, lower) of BALB/c mice (n=5 mice per group). Cleavage assays were run in triplicates, data are shown as mean ± s.e.m.. Normalized cleavage rates determined from the slope of the curves at the beginning of a reaction are displayed in Fig. 4c.

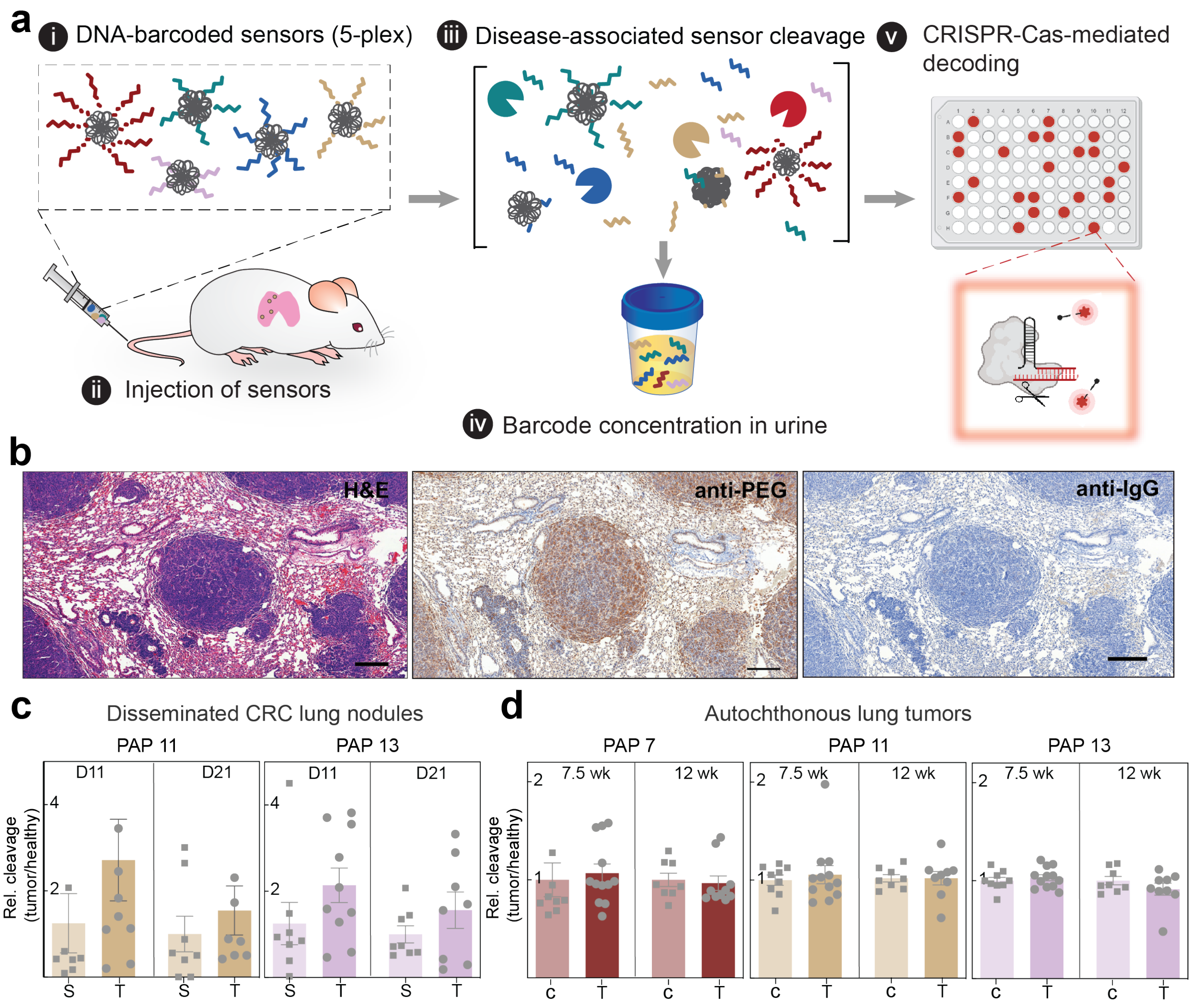

Supplementary Figure 9. DNA-encoded synthetic urinary biomarkers for multiplexed disease monitoring.

(**a**) Scheme of the workflow for longitudinal disease monitoring with the multiplexed DNA-encoded synthetic urinary biomarkers. (**b**) Histological (left) and immunohistochemistry staining of lung sections of BALB/c mice bearing CRC lung tumor nodules with anti-PEG (middle) and epitope control (right) antibody. (**c**) 5-plex DNA-SUBs were pooled and administered to BALB/c mice bearing CRC lung tumor nodules (T) and control animals (Sham, S) at day 11 or 21 after tumor initiation. All urine samples were collected at 1 h after sensor administration. Two sensors (DNA-PAP11-SUB, DNA-PAP13-SUB) showed an increase in the sets of tumor-bearing mice generated urine signals that were elevated relative to control animals (day 11, n=10 mice per tumor group; day 21, n=8 mice per tumor group; n=8 mice per control group; mean ± s.e.m.; no statistical significance in unpaired two-tailed *t*-test with Welch's correction). (**d**) 5-plex DNA-SUBs tested in KP lung tumor bearing (T) and healthy control (C) animals at 7.5 and 12 weeks after tumor initiation. Relative cleavage of three sensors (DNA-PAP7-SUB, DNA-PAP11-SUB, DNA-PAP13-SUB) relative to control animals (7.5 weeks, n=12 mice per tumor group; n=9 mice per control group; 12 weeks n=9 mice per tumor group; n=8 mice per control group; mean ± s.e.m.; no statistical significance in unpaired two-tailed *t*-test with Welch's correction).

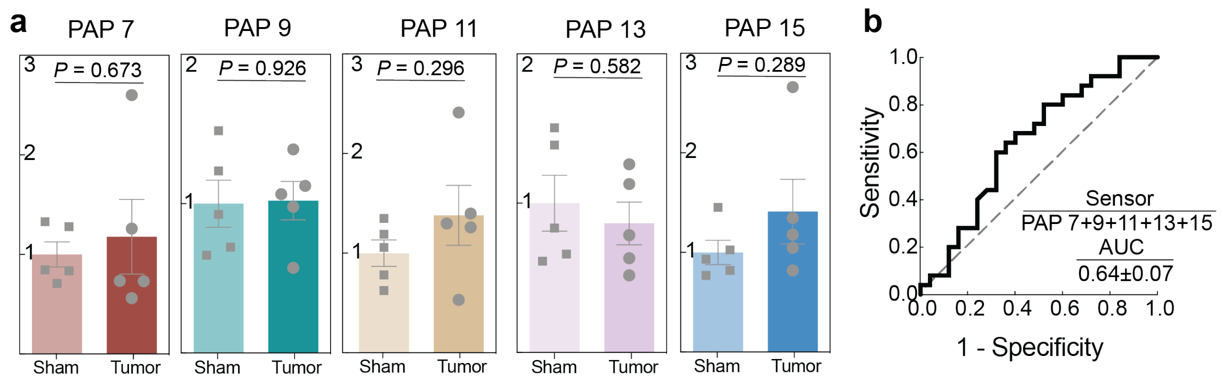

Supplementary Figure 10. Specificity of multiplexed DNA-encoded synthetic urinary biomarkers tested in PC-3 derived prostate cancer.

(**a**) 5-plex DNA-SUBs were pooled and administered to nude mice bearing subcutaneous PC-3 tumors. All urine samples were collected at 1 h after sensor administration. Three sensors (DNA-PAP7-SUB, DNA-PAP11-SUB, DNA-PAP15-SUB) showed an increase in the sets of tumor-bearing mice generated urine signals relative to healthy control animals (n=5 mice per tumor group; n=5 mice per control group; mean ± s.e.m.) No statistical significance was observed (unpaired two-tailed *t*-test with Welch's correction). (**b**) ROC analysis of the 5-plex DNA-SUBs to differentiate tumor from healthy control animals. The area under the curve (AUC) was calculated. Dashed line represents an AUC of 0.5, and a perfect AUC is 1.0.

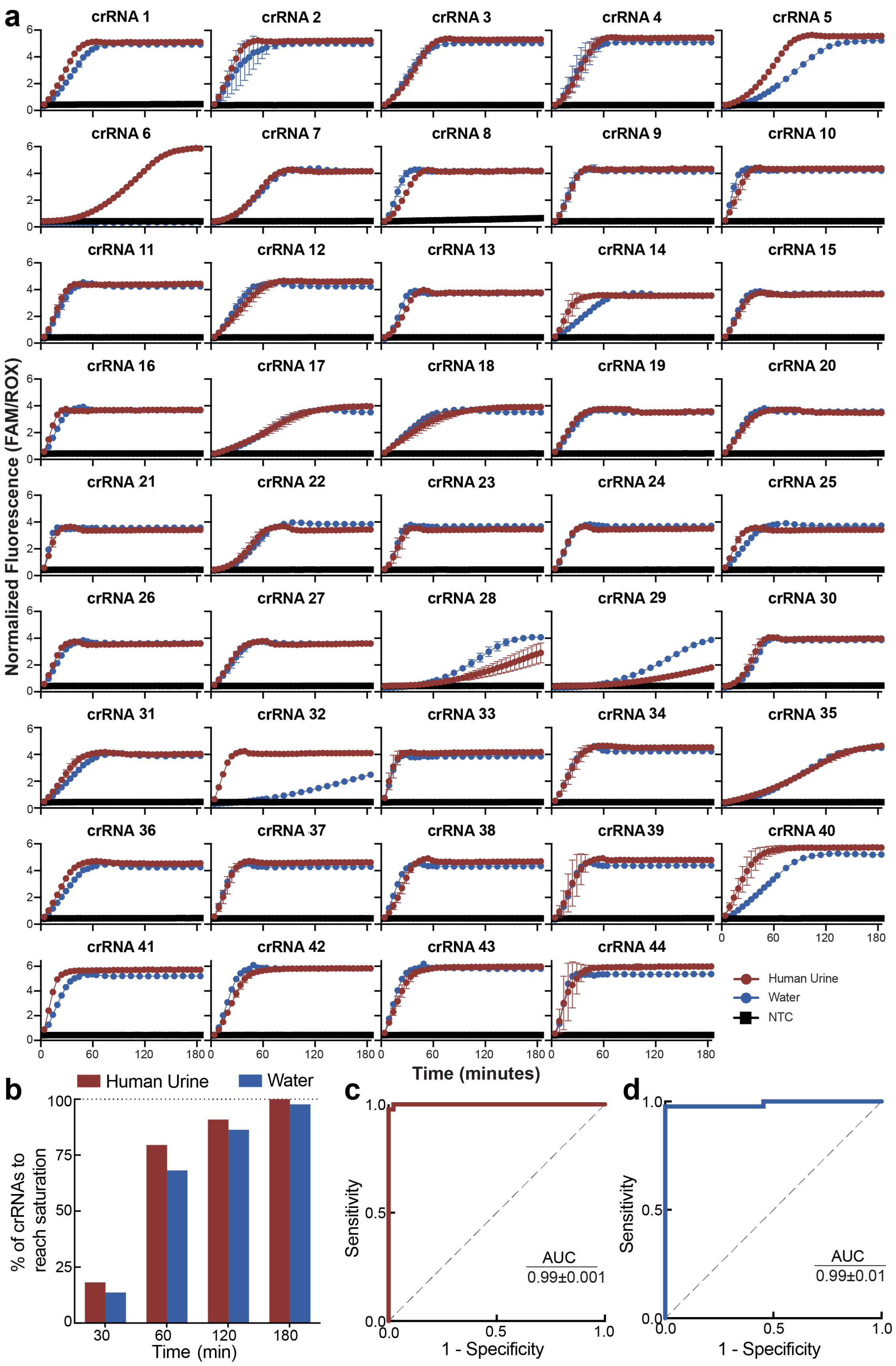
a
crRNA 1
crRNA 2
crRNA 3
crRNA 4
crRNA 5
crRNA 6
crRNA 7
crRNA 8
crRNA 9
crRNA 10
crRNA 11
crRNA 12
crRNA 13
crRNA 14
crRNA 15
crRNA 16
crRNA 17
crRNA 18
crRNA 19
crRNA 20
crRNA 21
crRNA 22
crRNA 23
crRNA 24
crRNA 25
crRNA 26
crRNA 27
crRNA 28
crRNA 29
crRNA 30
crRNA 31
crRNA 32
crRNA 33
crRNA 34
crRNA 35
crRNA 36
crRNA 37
crRNA 38
crRNA 39
crRNA 40
crRNA 41
crRNA 42
crRNA 43
crRNA 44
Normalized Fluorescence (FAM/ROX)
Time (minutes)
Human Urine
Water
NTC
b
Human Urine
Water
% of crRNAs to reach saturation
Time (min)
c
Sensitivity
1 - Specificity
AUC
0.99±0.001
d
Sensitivity
1 - Specificity
AUC
0.99±0.01

Supplementary Figure 11. Massively in-parallel CRISPR-Cas-mediated DNA detection on the Fluidigm microfluidics platform.

(**a**) Testing a comprehensive 20-mer DNA activator and crRNA panel using Fluidigm microfluidic platform in Fig. 5g. 44 synthetic 20-mer phosphorothioate-modified ssDNAs and their complementary crRNAs with 2 non-targeting pairs were spiked in human urine and water, respectively. Normalized fluorescence signal (FAM/ROX) were determined. Black lines, off-target ssDNA-crRNAs pairs tested (n=2 independent wells on microfluidic chip, data are shown as mean ± s.e.m.). (**b**) Percentage of ssDNA-crRNA pairs reach saturation in the duration of 3 h on the Fluidigm microfluidic chip. AUCs for ROCs for on-target versus off-target reactivity for samples spiked in human urine (**c**) and water (**d**). Dashed line represents an AUC of 0.5, and a perfect AUC is 1.0. Sequences of oligonucleotides were shown in Supplementary Table 1.

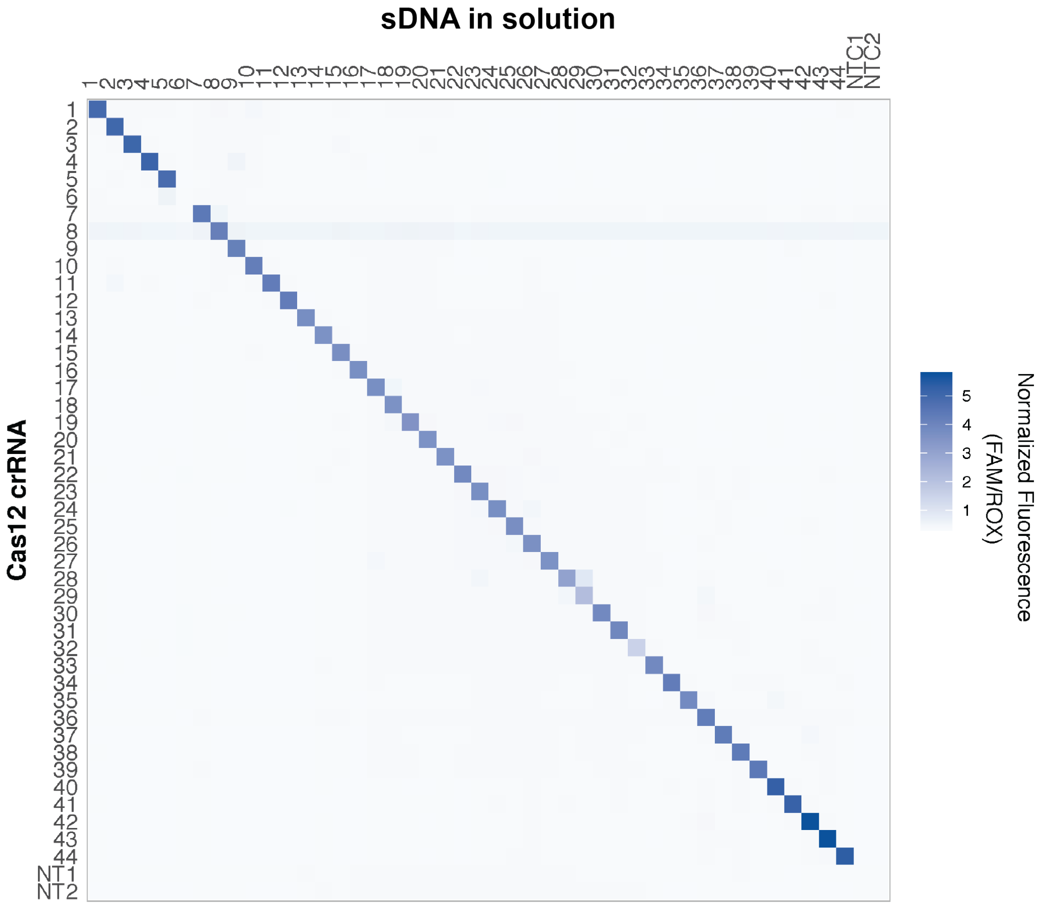

Supplementary Figure 12. Massively in-parallel CRISPR-Cas-mediated DNA detection in solution on the Fluidigm microfluidics platform.

Heatmap of *trans*-cleavage rates of different modified ssDNA activator-crRNA pairs. ssDNAs were dissolved in nuclease-free water and assays were performed in solution on a Fludigm microfluidic platform.

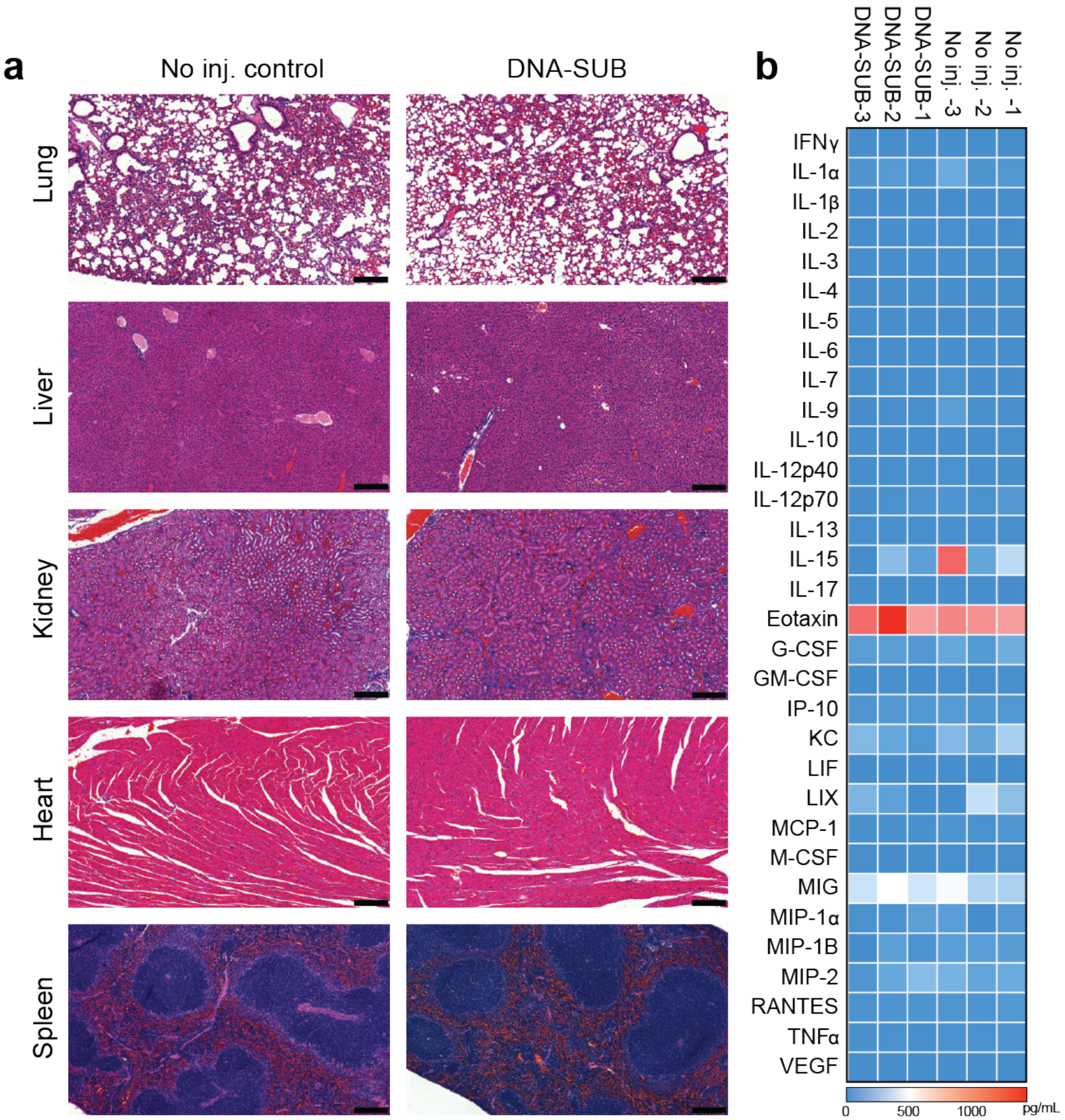

Supplementary Figure 13. ***In vivo*** **toxicity of DNA-encoded synthetic urinary biomarkers.** (**a**). Immunocompetent BALB/c mice were intravenously injected with multiplexed sensors at the same dose as in Fig 4. Organs (lung, liver, kidney, heart and spleen) were collected after 1 week of sensor administration. Organs were fixed, embedded in paraffin, and stained with hematoxylin and eosin. Study was done with n=3 mice per time point and images from a representative animal are shown. Scale bar = 200 µm. (**b**) Blood samples collected from mice with or without sensor injection (n=3 mice per group) were analyzed with the mouse Cytokine/Chemokine 32-plex array (Eve Technologies). Legend, numeric concentration of assayed proteins (pg/mL).

Supplementary Table 1. Nucleic acid sequences used in this study.

Name of oligo	Sequence (5'→ 3')
crRNA 1	rUrArA rUrUrU rCrUrA rCrUrA rArGrU rGrUrA rGrArU rCrGrUrCrGrCrCrGrUrCrCrArGrCrUrCrGrArCrC
crRNA 2	rUrArA rUrUrU rCrUrA rCrUrA rArGrU rGrUrA rGrArU rGrArUrCrGrUrUrArCrGrCrUrArArCrUrArUrGrA
crRNA 3	rUrArA rUrUrU rCrUrA rCrUrA rArGrU rGrUrA rGrArU rCrCrUrGrGrGrUrGrUrUrCrCrArCrArGrCrUrGrA
crRNA 5	rUrArA rUrUrU rCrUrA rCrUrA rArGrU rGrUrA rGrArU rCrTrGrTrGrTrTrTrArTrCrCrGrCrUrCrArCrArA
crRNA 6	rUrArA rUrUrU rCrUrA rCrUrA rArGrU rGrUrA rGrArU rUrGrArArGrUrArGrArUrArUrGrGrCrArGrCrArC
crRNA 7	rUrArA rUrUrU rCrUrA rCrUrA rArGrU rGrUrA rGrArU rArCrArArUrArUrGrUrGrCrUrUrCrUrArCrArCrA
ssDNA	TAGCATTCCACAGACAGCCCTCATAGTTAGCGTAACGATCTAAA GTTTTGTCGTC
Mod ssDNA	T*A*G*C*A*T*T*C*C*A*C*A*G*A*C*A*G*C*C*C*T*C*A*T*A*G*T*T* A*G*C*G*T*A*A*C*G*A*T*C*T*A*A*A*G*T*T*T*T*G*T*C*G*T*C
dsDNA- strand 1	TAGCATTCCACAGACAGCCCTCATAGTTAGCGTAACGATCTAAA GTTTTGTCGTC
dsDNA- strand 2 (complementary)	GACGACAAAACTTTAGATCGTTACGCTAACTATGAGGGCTGTCT GTGGAATGCTA
Mod DNA 1	G*G*T*C*G*A*G*C*T*G*G*A*C*G*G*C*G*A*C*G
Mod DNA 2	T*C*A*T*A*G*T*T*A*G*C*G*T*A*A*C*G*A*T*C
Mod DNA 3	T*C*A*G*C*T*G*T*G*G*A*A*C*A*C*C*C*A*G*G
Mod DNA 4	G*A*G*T*A*A*C*A*G*A*C*A*T*G*G*A*C*C*A*T*C*A*G
Mod DNA 5	T*T*G*T*G*A*G*C*G*G*A*T*A*A*A*C*A*C*A*G
Mod DNA 6	G*T*G*C*T*G*C*C*A*T*A*T*C*T*A*C*T*T*C*A
Mod DNA 7	T*G*T*G*T*A*G*A*A*G*C*A*C*A*T*A*T*T*G*T
Dye- DNA 2	/Cy5/TCATAGTTAGCGTAACGATC
Dye- Mod DNA 2	/Cy5/T*C*A*T*A*G*T*T*A*G*C*G*T*A*A*C*G*A*T*C
10 mer	CGTAACGATC
15 mer	GTTAGCGTAACGATC
18 mer	ATAGTTAGCGTAACGATC
20 mer	TCATAGTTAGCGTAACGATC
22 mer	TCATAGTTAGCGTAACGATCTA
24 mer	TCATAGTTAGCGTAACGATCTAAA
26 mer	CAGCCCTCATAGTTAGCGTAACGATC
30 mer	CAGCCCTCATAGTTAGCGTAACGATCTAAA
34 mer	GACAGCCCTCATAGTTAGCGTAACGATCTAAAGT
Mod 10 mer	C*G*T*A*A*C*G*A*T*C
Mod 15 mer	G*T*T*A*G*C*G*T*A*A*C*G*A*T*C
Mod 18 mer	A*T*A*G*T*T*A*G*C*G*T*A*A*C*G*A*T*C
Mod 20 mer	T*C*A*T*A*G*T*T*A*G*C*G*T*A*A*C*G*A*T*C
Mod 22 mer	T*C*A*T*A*G*T*T*A*G*C*G*T*A*A*C*G*A*T*C*T*A
Mod 24 mer	T*C*A*T*A*G*T*T*A*G*C*G*T*A*A*C*G*A*T*C*T*A*A*A
Mod 26 mer	C*A*G*C*C*C*T*C*A*T*A*G*T*T*A*G*C*G*T*A*A*C*G*A*T*C
Mod 30 mer	C*A*G*C*C*C*T*C*A*T*A*G*T*T*A*G*C*G*T*A*A*C*G*A*T*C*T*A* A*A

Mod 34 mer	G*A*C*A*G*C*C*C*T*C*A*T*A*G*T*T*A*G*C*G*T*A*A*C*G*A*T*C*T*A*A*A*G*T
Mod DNA 1 3'-DBCO	G*G*T*C*G*A*G*C*T*G*G*A*C*G*G*C*G*A*C*G\DBCO
Mod DNA 2 3'-DBCO	T*C*A*T*A*G*T*T*A*G*C*G*T*A*A*C*G*A*T*C\DBCO
Mod DNA 3 3'-DBCO	T*C*A*G*C*T*G*T*G*G*A*A*C*A*C*C*C*A*G*G\DBCO
Mod DNA 4 3'-DBCO	G*A*G*T*A*A*C*A*G*A*C*A*T*G*G*A*C*C*A*T*C*A*G\DBCO
Mod DNA 5 3'-DBCO	T*T*G*T*G*A*G*C*G*G*A*T*A*A*A*C*A*C*A*G\DBCO
Mod DNA 6 3'-DBCO	G*T*G*C*T*G*C*C*A*T*A*T*C*T*A*C*T*T*C*A\DBCO
Mod DNA 7 3'-DBCO	T*G*T*G*T*A*G*A*A*G*C*A*C*A*T*A*T*T*G*T\DBCO
Mod DNA 2 3'- Azide	T*C*A*T*A*G*T*T*A*G*C*G*T*A*A*C*G*A*T*C\Azide
Fluidigm_crRNA_1	rUrArA rUrUrU rCrUrA rCrUrA rArGrU rGrUrA rGrArU rArUrC rArUrU rUrArA rCrCrA rUrCrU rUrUrA rArCrC
Fluidigm_crRNA_2	rUrArA rUrUrU rCrUrA rCrUrA rArGrU rGrUrA rGrArU rArGrA rUrArG rGrGrA rCrUrC rUrUrA rArCrA rUrCrC
Fluidigm_crRNA_3	rUrArA rUrUrU rCrUrA rCrUrA rArGrU rGrUrA rGrArU rUrUrG rGrGrG rUrArU rGrGrG rGrGrG rGrGrC rGrGrU
Fluidigm_crRNA_4	rUrArA rUrUrU rCrUrA rCrUrA rArGrU rGrUrA rGrArU rCrCrG rUrArU rUrArU rArCrG rCrGrA rUrUrA rArCrG
Fluidigm_crRNA_5	rUrArA rUrUrU rCrUrA rCrUrA rArGrU rGrUrA rGrArU rCrGrC rGrArC rUrArU rArCrG rCrGrC rArArU rArUrG
Fluidigm_crRNA_6	rUrArA rUrUrU rCrUrA rCrUrA rArGrU rGrUrA rGrArU rArUrA rCrGrC rGrCrG rArCrU rArUrA rCrGrC rGrCrC
Fluidigm_crRNA_7	rUrArA rUrUrU rCrUrA rCrUrA rArGrU rGrUrA rGrArU rCrGrU rCrGrC rCrGrU rCrCrA rGrCrU rCrGrA rCrC
Fluidigm_crRNA_8	rUrArA rUrUrU rCrUrA rCrUrA rArGrU rGrUrA rGrArU rGrArU rCrGrU rUrArC rGrCrU rArArC rUrArU rGrA
Fluidigm_crRNA_9	rUrArA rUrUrU rCrUrA rCrUrA rArGrU rGrUrA rGrArU rCrCrU rGrGrG rUrGrU rUrCrC rArCrA rGrCrU rGrA
Fluidigm_crRNA_10	rUrArA rUrUrU rCrUrA rCrUrA rArGrU rGrUrA rGrArU rCrUrG rArUrG rGrUrC rCrArU rGrUrC rUrGrU rUrA
Fluidigm_crRNA_11	rUrArA rUrUrU rCrUrA rCrUrA rArGrU rGrUrA rGrArU rCrUrG rUrGrU rUrUrA rUrCrC rGrCrU rCrArC rArA
Fluidigm_crRNA_12	rUrArA rUrUrU rCrUrA rCrUrA rArGrU rGrUrA rGrArU rUrGrA rArGrU rArGrA rUrArU rGrGrC rArGrC rArC
Fluidigm_crRNA_13	rUrArA rUrUrU rCrUrA rCrUrA rArGrU rGrUrA rGrArU rArCrA rArUrA rUrGrU rGrCrU rUrCrU rArCrA rCrA
Fluidigm_crRNA_14	rUrArA rUrUrU rCrUrA rCrUrA rArGrU rGrUrA rGrArU rCrCrA rCrUrC rArCrU rGrCrU rUrUrC rUrCrC rUrC
Fluidigm_crRNA_15	rUrArA rUrUrU rCrUrA rCrUrA rArGrU rGrUrA rGrArU rUrUrU rCrCrC rUrUrC rArGrC rUrArA rArArU rArA
Fluidigm_crRNA_16	rUrArA rUrUrU rCrUrA rCrUrA rArGrU rGrUrA rGrArU rGrUrC rGrGrC rArUrG rGrCrC rCrCrA rUrUrC rGrC
Fluidigm_crRNA_17	rUrArA rUrUrU rCrUrA rCrUrA rArGrU rGrUrA rGrArU rArCrA rArUrA rUrGrU rGrCrU rUrCrU rArCrA rCrA
Fluidigm_crRNA_18	rUrArA rUrUrU rCrUrA rCrUrA rArGrU rGrUrA rGrArU rArGrA rUrArG rGrGrA rCrUrC rUrUrA rArCrA rUrCrC
Fluidigm_crRNA_19	rUrArA rUrUrU rCrUrA rCrUrA rArGrU rGrUrA rGrArU rUrUrA rArUrU rUrGrU rUrArC rGrGrA rArUrC rArU
Fluidigm_crRNA_20	rUrArA rUrUrU rCrUrA rCrUrA rArGrU rGrUrA rGrArU rCrArA rCrGrC rGrGrA rCrGrG rGrArA rArArC rCrU

Fluidigm_crRNA_21	rUrArA rUrUrU rCrUrA rCrUrA rArGrU rGrUrA rGrArU rArArC rGrArA rCrCrA rCrCrA rGrCrA rGrArA rGrA
Fluidigm_crRNA_22	rUrArA rUrUrU rCrUrA rCrUrA rArGrU rGrUrA rGrArU rCrUrA rCrArU rUrArC rArGrG rCrUrA rArCrA rArA
Fluidigm_crRNA_23	rUrArA rUrUrU rCrUrA rCrUrA rArGrU rGrUrA rGrArU rGrUrA rCrArU rUrGrC rArArG rArUrA rCrUrA rArA
Fluidigm_crRNA_24	rUrArA rUrUrU rCrUrA rCrUrA rArGrU rGrUrA rGrArU rArUrU rCrUrC rCrGrA rArCrG rUrGrU rCrArC rGrU
Fluidigm_crRNA_25	rUrArA rUrUrU rCrUrA rCrUrA rArGrU rGrUrA rGrArU rUrGrU rArCrU rArCrA rCrArA rArArG rUrArC rUrG
Fluidigm_crRNA_26	rUrArA rUrUrU rCrUrA rCrUrA rArGrU rGrUrA rGrArU rUrCrU rCrCrG rArArC rGrUrG rUrCrA rCrGrU rUrU
Fluidigm_crRNA_27	rUrArA rUrUrU rCrUrA rCrUrA rArGrU rGrUrA rGrArU rCrGrU rGrArC rArCrG rUrUrC rGrGrA rGrArA rUrU
Fluidigm_crRNA_28	rUrArA rUrUrU rCrUrA rCrUrA rArGrU rGrUrA rGrArU rGrCrU rUrGrC rUrGrU rCrCrC rUrGrU rUrGrU rUrC
Fluidigm_crRNA_29	rUrArA rUrUrU rCrUrA rCrUrA rArGrU rGrUrA rGrArU rArArU rArGrG rUrArG rArCrA rUrCrU rArArG rUrA
Fluidigm_crRNA_30	rUrArA rUrUrU rCrUrA rCrUrA rArGrU rGrUrA rGrArU rArArU rArCrU rUrArG rArUrG rUrCrU rArCrC rUrA
Fluidigm_crRNA_31	rUrArA rUrUrU rCrUrA rCrUrA rArGrU rGrUrA rGrArU rArUrU rGrUrU rCrGrU rArUrA rCrGrA rArCrG rUrA
Fluidigm_crRNA_32	rUrArA rUrUrU rCrUrA rCrUrA rArGrU rGrUrA rGrArU rArUrC rGrUrU rCrGrU rArUrA rCrGrA rArCrA rUrA
Fluidigm_crRNA_33	rUrArA rUrUrU rCrUrA rCrUrA rArGrU rGrUrA rGrArU rArUrC rArCrU rArUrC rArGrU rGrArU rArGrA rGrA
Fluidigm_crRNA_34	rUrArA rUrUrU rCrUrA rCrUrA rArGrU rGrUrA rGrArU rUrArC rUrGrA rUrArG rUrGrA rCrArA rArUrG rArC
Fluidigm_crRNA_35	rUrArA rUrUrU rCrUrA rCrUrA rArGrU rGrUrA rGrArU rUrCrU rArGrA rUrCrG rGrUrG rUrArA rArGrA rUrU
Fluidigm_crRNA_36	rUrArA rUrUrU rCrUrA rCrUrA rArGrU rGrUrA rGrArU rGrGrG rUrArA rUrCrU rUrUrG rCrArU rUrCrG rArU
Fluidigm_crRNA_37	rUrArA rUrUrU rCrUrA rCrUrA rArGrU rGrUrA rGrArU rArUrC rCrUrA rGrGrU rCrGrU rGrArU rUrCrA rUrA
Fluidigm_crRNA_38	rUrArA rUrUrU rCrUrA rCrUrA rArGrU rGrUrA rGrArU rCrUrU rUrUrU rUrUrU rArGrG rGrCrG rArCrG rUrU
Fluidigm_crRNA_39	rUrArA rUrUrU rCrUrA rCrUrA rArGrU rGrUrA rGrArU rArUrG rArArG rArGrA rGrUrU rArCrA rGrArA rArU
Fluidigm_crRNA_40	rUrArA rUrUrU rCrUrA rCrUrA rArGrU rGrUrA rGrArU rUrUrC rGrArU rArCrA rCrCrA rGrArC rUrGrC rUrA
Fluidigm_crRNA_41	rUrArA rUrUrU rCrUrA rCrUrA rArGrU rGrUrA rGrArU rArGrG rArArG rGrCrU rArGrU rCrUrU rGrUrA rCrU
Fluidigm_crRNA_42	rUrArA rUrUrU rCrUrA rCrUrA rArGrU rGrUrA rGrArU rArUrA rGrGrU rCrCrC rUrArU rArArG rArCrA rUrA
Fluidigm_crRNA_43	rUrArA rUrUrU rCrUrA rCrUrA rArGrU rGrUrA rGrArU rUrArG rUrUrU rGrArC rArArG rCrArG rArUrA rArC
Fluidigm_crRNA_44	rUrArA rUrUrU rCrUrA rCrUrA rArGrU rGrUrA rGrArU rGrUrA rGrUrU rUrUrA rGrGrU rUrCrU rGrGrA rGrA

Fluidigm_Barcode_1	G*G*T*T*A*A*A*G*A*T*G*G*T*T*A*A*A*T*G*A*T
Fluidigm_Barcode_2	G*G*A*T*G*T*T*A*A*G*A*G*T*C*C*C*T*A*T*C*T
Fluidigm_Barcode_3	A*C*C*G*C*C*C*C*C*C*C*C*C*A*T*A*C*C*C*C*A*A
Fluidigm_Barcode_4	C*G*T*T*A*A*T*C*G*C*G*T*A*T*A*A*T*A*C*G*G
Fluidigm_Barcode_5	C*A*T*A*T*T*G*C*G*C*G*T*A*T*A*G*T*C*G*C*G
Fluidigm_Barcode_6	G*G*C*G*C*G*T*A*T*A*G*T*C*G*C*G*C*G*T*A*T
Fluidigm_Barcode_7	G*G*T*C*G*A*G*C*T*G*G*A*C*G*G*C*G*A*C*G
Fluidigm_Barcode_8	T*C*A*T*A*G*T*T*A*G*C*G*T*A*A*C*G*A*T*C
Fluidigm_Barcode_9	T*C*A*G*C*T*G*T*G*G*A*A*C*A*C*C*C*A*G*G
Fluidigm_Barcode_10	T*A*A*C*A*G*A*C*A*T*G*G*A*C*C*A*T*C*A*G
Fluidigm_Barcode_11	T*T*G*T*G*A*G*C*G*G*A*T*A*A*A*C*A*C*A*G
Fluidigm_Barcode_12	G*T*G*C*T*G*C*C*A*T*A*T*C*T*A*C*T*T*C*A
Fluidigm_Barcode_13	T*G*T*G*T*A*G*A*A*G*C*A*C*A*T*A*T*T*G*T
Fluidigm_Barcode_14	G*A*G*G*A*G*A*A*A*G*C*A*G*T*G*A*G*T*G*G
Fluidigm_Barcode_15	T*T*A*T*T*T*T*A*G*C*T*G*A*A*G*G*G*A*A*A
Fluidigm_Barcode_16	G*C*G*A*A*T*G*G*G*G*C*C*A*T*G*C*C*G*A*C
Fluidigm_Barcode_17	G*A*T*G*C*C*G*T*T*C*T*T*C*T*G*C*T*T*G*T
Fluidigm_Barcode_18	C*A*G*G*T*A*A*A*C*A*C*A*C*A*A*A*C*C*T*T
Fluidigm_Barcode_19	G*A*T*T*C*C*G*T*A*A*C*A*A*A*T*T*A*A
Fluidigm_Barcode_20	T*T*T*T*C*C*C*G*T*C*C*G*C*G*T*T*G
Fluidigm_Barcode_21	T*C*T*T*C*T*G*C*T*G*G*T*G*G*T*T*C*G*T*T
Fluidigm_Barcode_22	T*T*T*G*T*T*A*G*C*C*T*G*T*A*A*T*G*T*A*G
Fluidigm_Barcode_23	T*T*T*A*G*T*A*T*C*T*T*G*C*A*A*T*G*T*A*C
Fluidigm_Barcode_24	A*C*G*T*G*A*C*A*C*G*T*T*C*G*G*A*G*A*A*T
Fluidigm_Barcode_25	C*A*G*T*A*C*T*T*T*T*G*T*G*T*A*G*T*A*C*A
Fluidigm_Barcode_26	A*A*A*C*G*T*G*A*C*A*C*G*T*T*C*G*G*A*G*A
Fluidigm_Barcode_27	A*A*T*T*C*T*C*C*G*A*A*C*G*T*G*T*C*A*C*G
Fluidigm_Barcode_28	G*A*A*C*A*A*C*A*G*G*G*A*C*A*G*C*A*A*G*C
Fluidigm_Barcode_29	T*A*C*T*T*A*G*A*T*G*T*C*T*A*C*C*T*A*T*T
Fluidigm_Barcode_30	T*A*G*G*T*A*G*A*C*A*T*C*T*A*A*G*T*A*T*T
Fluidigm_Barcode_31	T*A*C*G*T*T*C*G*T*A*T*A*C*G*A*A*C*A*A*T
Fluidigm_Barcode_32	T*A*T*G*T*T*C*G*T*A*T*A*C*G*A*A*C*G*A*T
Fluidigm_Barcode_33	T*C*T*C*T*A*T*C*A*C*T*G*A*T*A*G*T*G*A*T
Fluidigm_Barcode_34	G*T*C*A*T*T*T*G*T*C*A*C*T*A*T*C*A*G*T*A
Fluidigm_Barcode_35	A*A*T*C*T*T*T*A*C*A*C*C*G*A*T*C*T*A*G*A

Fluidigm_Barcode_36	A*T*C*G*A*A*T*G*C*A*A*A*G*A*T*T*A*C*C*C
Fluidigm_Barcode_37	T*A*T*G*A*A*T*C*A*C*G*A*C*C*T*A*G*G*A*T
Fluidigm_Barcode_38	A*A*C*G*T*C*G*C*C*C*T*A*A*A*A*A*A*A*A*G
Fluidigm_Barcode_39	A*T*T*T*C*T*G*T*A*A*C*T*C*T*C*T*T*C*A*T
Fluidigm_Barcode_40	T*A*G*C*A*G*T*C*T*G*G*T*G*T*A*T*C*G*A*A
Fluidigm_Barcode_41	A*G*T*A*C*A*A*G*A*C*T*A*G*C*C*T*T*C*C*T
Fluidigm_Barcode_42	T*A*T*G*T*C*T*T*A*T*A*G*G*G*A*C*C*T*A*T
Fluidigm_Barcode_43	G*T*T*A*T*C*T*G*C*T*T*G*T*C*A*A*A*C*T*A
Fluidigm_Barcode_44	T*C*T*C*C*A*G*A*A*C*C*T*A*A*A*A*C*T*A*C

*, phosphorothioate modification
DBCO, Dibenzocyclooctyne
Cy5, Cyanine 5 dye

Supplementary Table 2. Activation of Cas12a with native and modified DNA oligos *in vitro* and *in vivo.*

Activation of *Lba*Cas12a with native and modified DNA oligos were quantified in the Cas12a fluorescent cleavage assay. For 'DNA *in vitro*', 4 nM of DNA activators with different length were added in each reaction. For 'DNA *in vivo*', 1 nmol of DNA activators, native or modified, with different length were injected into healthy BALB/c mice and urine samples collected after 1 h of injection were added in each reaction. *The initial reaction velocity (V_0) refers to the slope of the curve at the beginning of a reaction.

Oligo	Modified DNA *in vitro* (V_0)*	Modified DNA *in vivo* (V_0)	Native DNA *in vitro* (V_0)	Native DNA *in vivo* (V_0)
10 mer	0.01	0.01	0.00	0.00
15 mer	0.01	0.01	0.00	0.00
18 mer	3.65	0.61	5.03	0.00
20 mer	6.29	1.82	7.67	0.02
24 mer	8.91	0.92	10.94	0.00
26 mer	5.25	0.60	6.09	0.01
30 mer	4.65	0.34	4.98	0.00
34 mer	4.55	0.38	3.32	0.01

Supplementary Table 3. Peptide and protein sequences used in this study.

Name of peptide	Sequence (N→ C)
PAP7-click	K(N3)-ANP-GGPLGVRGKGGC
PAP9-click	K(N3)-ANP-GGfPRSGGC
PAP11-click	K(N3)-ANP-GGGSGRSANAKGGC
PAP13-click	K(N3)-ANP-GGVPRGGC
PAP15-click	K(N3)-ANP-GPVPLSLVMGGC
FRET-PAP1	5FAM-GGPQGIWGQK(CPQ2)-PEG2-GC
FRET-PAP2	5FAM-GGLVPRGSGK(CPQ2)-PEG2-GC
FRET-PAP3	5FAM-GGPVGLIGK(CPQ2)-PEG2-GC
FRET-PAP4	5FAM-GGPWGIWGQGK(CPQ2)-PEG2-GC
FRET-PAP5	5FAM-GGPVPLSLVMK(CPQ2)-PEG2-GC
FRET-PAP6	5FAM-GGPLGLRSWK(CPQ2)-PEG2-GC
FRET-PAP7	5FAM-GGPLGVRGKK(CPQ2)-PEG2-GC
FRET-PAP8	5FAM-GGf-Pip-RSGGGK(CPQ2)-PEG2-GC
FRET-PAP9	5FAM-GGfPRSGGGK(CPQ2)-PEG2-GC
FRET-PAP10	5FAM-GGf-Pip-KSGGGK(CPQ2)-PEG2-GC
FRET-PAP11	5FAM-GGGSGRSANAKG-K(CPQ2)-PEG2-GC
FRET-PAP12	5FAM-GILSRIVGGG-K(CPQ2)-PEG2-GC
FRET-PAP13	5FAM-GGVPRGG-K(CPQ2)-PEG2-GC
FRET-PAP14	5FAM-GSGSKIIGGG-K(CPQ2)-PEG2-GC
FRET-PAP15	5FAM-GPVPLSLVMG-K(CPQ2)-PEG2-GC
FRET-PAP16	5FAM-GGLGPKGQTGK(CPQ2)-kk-PEG2-C
cMET nanobody	MEVQLVESGGGLVQPGGSLRLSCAASGFILDYYAIGWFRQAPGKEREGVLCIDASDDITYYADSVKGRFTISRDNAKNTVYLQMNSLKPEDTGVYYCATPIGLSSSCLLEYDYDYWGQGTLVTVSSGSHHHHHHSPSTPPTPSPSTPPGSGRSANAKGGGSC (Clone 4E09, sequence is available from https://patents.google.com/patent/WO2012042026A1/en)
GFP nanobody	MAQVQLVESGGRLVQAGDSLRLSCAASGRTFSTSAMAWFRQAPGREREFVAAITWTVGNTILGDSVKGRFTISRDRAKNTVDLQMDNLEPEDTAVYYCSARSRGYVLSVLRSVDSYDYWGQGTQVTVSGSHHHHHHSPSTPPTPSPSTPPGSGRSANAKGGGSC (Clone LaG-16) (Fridy et al., 2014)

Upper case, L-form amino acid; lower case, D-form amino acid;
N3, Azide side chain; Cy7, Cyanine 7;
ANP, photocleavable linker; 5FAM, N-terminal Fluorescein fluorophore

Supplementary Table 4. Sequences of DNA-barcoded synthetic urinary biomarkers.

Name of sensors	Sequence (nucleotides 5’→3’, peptides N→ C)
PAP7-DNA2	T*C*A*T*A*G*T*T*A*G*C*G*T*A*A*C*G*A*T*C-ANP-GGPLGVRGKGGC-PEG40k (maleimide)
PAP9-DNA1	G*G*T*C*G*A*G*C*T*G*G*A*C*G*G*C*G*A*C*G-ANP-GG-DPhe-PRSGGC-PEG40k (maleimide)
PAP11-DNA3	T*C*A*G*C*T*G*T*G*G*A*A*C*A*C*C*C*A*G*G-ANP-GGGSGRSANAKGGC- PEG40k (maleimide)
PAP13-DNA6	G*T*G*C*T*G*C*C*A*T*A*T*C*T*A*C*T*T*C*A-ANP-GGVPRGGC-PEG40k (maleimide)
PAP15-DNA5	T*T*G*T*G*A*G*C*G*G*A*T*A*A*A*C*A*C*A*G-ANP-GPVPLSLVMGGC-PEG40k (maleimide)
cMET nanobody-DNA	MEVQLVESGGGLVQPGGSLRLSCAASGFILDYYAIGWFRQAPGKEREGVLCIDASDDITYYADSVKGRFTISRDNAKNTVYLQMNSLKPEDTGVYYCATPIGLSSSCLLEYDYDYWGQGTLVTVSSGSHHHHHHSPSTPPTPSPSTPPGSGRSANAKGGGSC-(3’) C*T*A*G*C*A*A*T*G*C*G*A*T*T*G*A*T*A*C*T*(5’)
GFP nanobody-DNA	MAQVQLVESGGRLVQAGDSLRLSCAASGRTFSTSAMAWFRQAPGREREFVAAITWTVGNTILGDSVKGRFTISRDRAKNTVDLQMDNLEPEDTAVYYCSARSRGYVLSVLRSVDSYDYWGQGTQVTVSGSHHHHHHSPSTPPTPSPSTPPGSGRSANAKGGGSC-(3’) C*T*A*G*C*A*A*T*G*C*G*A*T*T*G*A*T*A*C*T*(5’)

lower case, D-form amino acid; N3, Azide side chain; Cy7, Cyanine 7
ANP, photocleavable linker;
*, phosphorothioate modification

Supplementary Table 5. List of all primary antibodies.

Antibody	Catalog#	Manufacturer	Application	Dilution
Cathepsin D	ab75852	Abcam	IF	1:100
MMP3	ab194717	Abcam	IF	1:200
MMP7	ab5706	Abcam	IF	1:100
MMP9	ab38898	Abcam	IF	1:200
PEG	ab190652	Abcam	IHC	1:200
PLAU	ab24121	Abcam	IHC	1:100
cMET	ab51067	Abcam	IHC	1:100
Cyanine	sc-166895	Santa Cruz	IF	1:100

IF: immunofluorescence

IHC: Immunohistochemistry

Supplementary Table 6. List of buffers for proteolytic cleavage assays.

Enzyme	Manufacturer	Buffer
MMPs	Enzo	50 mM TriS, 10 mM $CaCl_2$, 300 mM NaCl, 20 μM $ZnCl_2$, 0.02% Brij-35, 1% BSA, pH 7.5
ADAMs	Enzo	10 mM HEPES, 100 mM NaCl, 0.01% Brij-35, 1% BSA, pH 7.4
Cathepsin B	R&D	25 mM MES, 5 mM DTT, pH 5.0
Cathepsin D	R&D	0.1 M NaOAc, 0.2 M NaCl, pH 3.5
Cathepsin E	R&D	0.1 M NaOAc, 0.5 M NaCl, pH 3.5
Cathepsin K	Enzo	50 mM NaOAc, 1mM DTT, pH 5.5
Cathepsin L	R&D	50 mM MES, 5 mM DTT, 1 mM EDTA, 0.005% (w/v) Brij-35, pH 6.0
Cathepsin S	R&D	50 mM NaOAc, 5 mM DTT, 250 mM NaCl, pH 4.5
uPA/PLAU	R&D	50 mM Tris, 0.01% Tween 20, 1% BSA, pH 7.4

Supplementary Code. Enzymatic kinetics analysis.

```
import pandas as pd
import numpy as np
from sklearn.linear_model import LinearRegression
import os
from itertools import islice


pd.options.mode.chained_assignment = None

#Convert Raw Data from Tecan Excel Sheets into individual CSV Files for each Tis
sue Sample
os.mkdir('./Kinetics')
excel_file=input("Enter name of Excel File ")
f=input("index of first Kinetics sheet ")
l=input("index of last Kinetics sheet ")
sheets_dict=pd.read_excel(excel_file, sheet_name=None)
for name,sheet in islice(sheets_dict.items(),int(f),int(l)+1):
    sheet.to_csv(os.path.join('./Kinetics', name + '.csv'), index= None, header
=False)

#Fold Change Calculations
os.mkdir('./FC')
f=input("index of first FC sheet ")
l=input("index of last FC sheet ")
for name,sheet in islice(sheets_dict.items(),int(f),int(l)+1):
    sheet.to_csv(os.path.join('./FC', name + '.csv'), index= None, header =False
)

#Fitting Linear Regression and calculating slope for each tissue
os.mkdir('./Rate')

a = input("Do you want to use the same number of time points for all tissues? (y
/n) ")
if a == "y":
    t=input("How many time points are you considering? ")

for filename in os.scandir('./Kinetics'):
    if(filename.path.endswith("csv")):
        name = os.path.splitext(os.path.basename(filename))[0]
        data=pd.read_csv(filename, header=None)
        av=np.zeros(shape=(len(data),int((len(data.columns)+2)/3)), dtype=object
)

        j=0
        for i in range(int((len(data.columns)+2)/3)):
            av[0][i]=data[j][0]
            j=j+3
```
