# Supplementary material for: CRISPR-Cas-amplified urinary biomarkers for multiplexed and portable cancer diagnostics
Source: Nat Nanotechnol. 2023 Apr 24;18(7):798–807. doi: 10.1038/s41565-023-01372-9 (PMC10359190; doi:10.1038/s41565-023-01372-9)
Supplement: Supplementary file 2 — Reporting Summary [file 41565_2023_1372_MOESM2_ESM.pdf]

nature portfolio

Corresponding author(s): Sangeeta N. Bhatia

Last updated by author(s): Feb 24, 2023

Reporting Summary

Nature Portfolio wishes to improve the reproducibility of the work that we publish. This form provides structure for consistency and transparency in reporting. For further information on Nature Portfolio policies, see our Editorial Policies and the Editorial Policy Checklist.

Statistics

For all statistical analyses, confirm that the following items are present in the figure legend, table legend, main text, or Methods section.

n/a	Confirmed	
☐	☒	The exact sample size (*n*) for each experimental group/condition, given as a discrete number and unit of measurement
☐	☒	A statement on whether measurements were taken from distinct samples or whether the same sample was measured repeatedly
☐	☒	The statistical test(s) used AND whether they are one- or two-sided<br>*Only common tests should be described solely by name; describe more complex techniques in the Methods section.*
☐	☒	A description of all covariates tested
☐	☒	A description of any assumptions or corrections, such as tests of normality and adjustment for multiple comparisons
☐	☒	A full description of the statistical parameters including central tendency (e.g. means) or other basic estimates (e.g. regression coefficient) AND variation (e.g. standard deviation) or associated estimates of uncertainty (e.g. confidence intervals)
☐	☒	For null hypothesis testing, the test statistic (e.g. *F*, *t*, *r*) with confidence intervals, effect sizes, degrees of freedom and *P* value noted<br>*Give P values as exact values whenever suitable.*
☒	☐	For Bayesian analysis, information on the choice of priors and Markov chain Monte Carlo settings
☒	☐	For hierarchical and complex designs, identification of the appropriate level for tests and full reporting of outcomes
☒	☐	Estimates of effect sizes (e.g. Cohen's *d*, Pearson's *r*), indicating how they were calculated

Our web collection on statistics for biologists contains articles on many of the points above.

Software and code

Policy information about availability of computer code

Data collection	Living Image (Version 4.5.5) was used to collect data on the IVIS® Spectrum in vivo imaging system (PerkinElmer Inc.). ImageStudio (Version 5.2) was used for the Odyssey CLx imaging system (Li-Cor Inc.). Tecan icontrol software (Version 3.7.3.0) was used to collect data on the Tecan Infinite 200pro microplate reader (Tecan Group Ltd.). Bio-Rad CFX manager 3.1 was used to collect data on the CFX96 Real Time System C1000 Thermal Cycler (Bio-Rad Laboratories Inc.). Panoramic Scanner (Version 1.22) was used to collect data on a 3DHistech P250 High Capacity Slide Scanner (PerkinElmer Inc.). ChemStation for LC 3D (Rev. B.03.01) was used to control the Agilent Model 1100 HPLC system (Agilent Technologies Inc.). UNICORN (Version 5.3.1) was used for FPLC on a AKTApurifier 10 (GE Healthcare Inc.). SoftMax Pro Version 4.8 was used for the SpectraMax Plus 384 Microplate Spectrophotometer (Molecular Devices LLC). Details are specified in Methods, Figure Legends and Supplementary Information.
Data analysis	Living Image (Version 4.7.3, Perkin Elmer Inc.) was used for IVIS in vivo optical imaging analysis. ImageStudio (Version 5.2) was used for analysis of near-infrared images collected on the Odyssey CLx imaging system (Li-Cor Inc.). CaseViewer (Version 2.2) was used for processing of images collected on the 3DHistech P250 High Capacity Slide Scanner (3DHISTECH Ltd.). ImageJ (1.49v) was used for lateral flow strip quantification. Differential expression analyses were carried out by DESeq2 1.10.1. GraphPad Prism (Version 9) was used for data analysis and statistics. Microfluidics readout on the Fluidigm BioMark HD System was analyzed using Python 3 custom analysis codes available on GitHub: https://github.com/broadinstitute/mcarmen. Python (version 3.9) package for enzyme kinetics analysis is available through GitHub: https://github.com/nharzallah/LMRT-NNanotech-2023. Details are specified in Methods, Figure Legends and Supplementary Information.

For manuscripts utilizing custom algorithms or software that are central to the research but not yet described in published literature, software must be made available to editors and reviewers. We strongly encourage code deposition in a community repository (e.g. GitHub). See the Nature Portfolio guidelines for submitting code & software for further information.

Data

Policy information about availability of data

All manuscripts must include a data availability statement. This statement should provide the following information, where applicable:

- Accession codes, unique identifiers, or web links for publicly available datasets
- A description of any restrictions on data availability
- For clinical datasets or third party data, please ensure that the statement adheres to our policy

The Cancer Genome Atlas (http://cancergenome.nih.gov) and Matrisome (http://matrisomeproject.mit.edu/) are open access resources. The datasets and codes analysed during the current study are available in the Zenodo repository (https://zenodo.org/deposit/7686811). All data that support the findings of this study are available within the Article and Supplementary Information or from the corresponding author upon reasonable request.

Field-specific reporting

Please select the one below that is the best fit for your research. If you are not sure, read the appropriate sections before making your selection.

☒ Life sciences ☐ Behavioural & social sciences ☐ Ecological, evolutionary & environmental sciences

For a reference copy of the document with all sections, see nature.com/documents/nr-reporting-summary-flat.pdf

Life sciences study design

All studies must disclose on these points even when the disclosure is negative.

Sample size	Sample sizes are provided in the figure legend for each experiment and were determined to be adequate based on the magnitude and consistency of measurable differences between groups. Samples were allowed to have at least three biological replicates to derive statistical significance. We used a sample size of minimum three mice per group for in vivo studies. Animal group sizes were also practically associated with the number of mice housed per cage (n=5 mice per cage). When comparison between two animal groups were conducted (e.g. comparing urinary barcode levels), sample size are selected to ensure that the number in one animal group is greater than or equal to five at each time point and for each treatment group, because a group size of four can provide 80% power to detect a 1.5-fold difference in urinary barcode levels (two-sided t-test, α set at 0.05). Numbers of animals per group were specified in the figure legends.
Data exclusions	No data was excluded for in vitro experiments. Pre-established exclusion criteria were set based on failed injection or urine production within defined time course of the animal study. Animals were excluded solely on the basis of the pre-established exclusion criteria.
Replication	All experiments were repeated independently at least twice with similar results. Experiments were repeated and visualized by two independent researchers when results from representative experiments (such as histological or fluorescent micrographs) are shown.
Randomization	In vitro samples were prepared, processed, and analyzed in a random order, for instance, DNA barcodes were randomly numbered. Cultured cells were randomly assigned to experimental groups. All animals analyzed in this study were sex- and age-matched. Littermates of the same sex were randomly assigned to experimental and control groups. Grouping criteria of animals was included in Methods and figure legends.
Blinding	For histological evaluation of toxicity, investigators were not blinded to group allocation during data collection but tissue sections were analyzed by a veterinary pathologist who was blinded to the treatment groups. In all other cases, investigators were not blinded to the groups and treatments during experiments, because the investigators who set up the experiments carried out the analyses. Data reported for these experiments are not subjective but based on the quantitative assays described in Methods and figure legends.

Reporting for specific materials, systems and methods

We require information from authors about some types of materials, experimental systems and methods used in many studies. Here, indicate whether each material, system or method listed is relevant to your study. If you are not sure if a list item applies to your research, read the appropriate section before selecting a response.

Materials & experimental systems

n/a	Involved in the study	
☐	☒	Antibodies
☐	☒	Eukaryotic cell lines
☒	☐	Palaeontology and archaeology
☐	☒	Animals and other organisms
☒	☐	Human research participants
☒	☐	Clinical data
☒	☐	Dual use research of concern

Methods

n/a	Involved in the study	
☒	☐	ChIP-seq
☒	☐	Flow cytometry
☒	☐	MRI-based neuroimaging

Antibodies

Antibodies used

Anti-Cathepsin D antibody [EPR3057Y], Abcam, Catalog # ab75852, Lot# GR260148-33;
anti-MMP3 antibody [EP1186Y] (Alexa Fluor® 647 conjugated), Abcam,Catalog# ab194717, Lot# GR203491-3;
anti-MMP7 antibody, Abcam, Catalog #ab5706, Lot# GR3345935-1;
anti-MMP9 antibody, Abcam, Catalog# ab58803, Lot# GR3212138-1;
anti-Polyethylene glycol antibody [RM105], Abcam, Catalog# ab190652, Lot# GR292148-5;
anti-PLAU antibody, Abcam, Catalog # ab24121, Lot# GR3273891-1;
anti-Met (c-Met) antibody, Abcam, catalog # ab51067, Lot# GR261314-24;
anti-Cyanine antibody (A-12), Santa Cruz Biotechnology, Catalog# sc-166936, Lot# J3111.
List of all primary antibodies and dilutions can be found in Supplementary Table 5.

Secondary antibodies:
Donkey anti-Rabbit IgG (H+L) Highly Cross-Adsorbed Secondary Antibody, Alexa Fluor® 647, ThermoFisher, Catalog# A-31573, Lot# 1322326;
Donkey anti-Mouse IgG (H+L) Highly Cross-Adsorbed Secondary Antibody, Alexa Fluor® 555, ThermoFisher, Catalog# A-31570, Lot# 412442;
Rabbit-on-Rodent HRP-Polymer (for use with rabbit monoclonal/polyclonal antibodies on mouse and rat tissue), Biocare Medical, Product # RMR 622.

Validation

All commercially available antibodies have been thoroughly tested and validated by the manufacture:

Anti-Cathepsin D antibody (ab75852, https://www.abcam.com/products/primary-antibodies/cathepsin-d-antibody-epr3057y-ab75852.html) has been validated in western blots with MCF7, A431, SK-BR-3 and HepG2 whole cell lysate and detected ~46 kDa. Tested applications also include IHC-P with human breast carcinoma and liver tissues; ICC/IF with MCF7 cells, IP with SK-BR-3 cell lysate, flow cytometry (intra) with HepG2 cells, and IHC-Fr with human liver tissue sections. It has been referenced in 72 publications. Selected recent citations:
Aoto K et al. Nat Commun 12:2107 (2021);
Lin J et al. Mol Med Rep 23:N/A (2021);
Wang X et al. Cell Death Dis 12:402 (2021).

Anti-MMP3 antibody (ab194717, https://www.abcam.com/products/primary-antibodies/alexa-fluor-647-mmp3-antibody-ep1186y-ab194717.html) was tested in ICC/IF with mouse NIH3T3 cells and the confocal image was available on the aforementioned manufacturer's website.

Anti-MMP7 antibody (ab5706, https://www.citeab.com/antibodies/739648-ab5706-anti-mmp7-antibody). This antibody was used in western blot knockout validation on mouse samples. Applications also include ELISA, ICC/IF, IHC-P, IP. It has been referenced in 72 publications. Selected recent citations:
Wang, Y. N. Front. Pharmacol. 13:964370 (2022);
Cao, J. Z. J Gastrointest Oncol 13:3 (2022);
Wu, Q., Mol. Ther. 29:3 (2021).

Anti-MMP9 antibody (ab38898, https://www.abcam.com/mmp9-antibody-ab38898.html) was shown to bind to the Recombinant Human MMP9, His tagged (ab82955) and also to the Recombinant Mouse MMP9 protein (ab39309) indicating that it is specific for the MMP9 target. ab38898 detects recombinant Human MMP9 running at ~85 kDa, and endogenous full-length MMP9 in LPS-stimulated cells at ~100 kDa. This antibody also detects a band at 90 kDa in U937 PMA-treated cells. Tested applications of the antibody include IHC-Fr, WB. This antibody has been referenced in 814 publications. Selected recent citations:
Qin T et al. J Ethnopharmacol 246:112128 (2020);
Chen X et al. J Exp Clin Cancer Res 39:65 (2020);
Hu L et al. J Gastric Cancer 20:95-105 (2020).

Anti-Polyethylene glycol antibody(ab190652, https://www.abcam.com/products/primary-antibodies/polyethylene-glycol-antibody-rm105-ab190652.html?productWallTab=ShowAll). This antibody has been tested with mouse liver and kidney tissue injected with PEG-BSA and PEGylated BSA. Tested applications include western blot and ELISA with PEGylated BSA, and IHC with mouse liver and kidney tissue labeling PEG. A recent citation:
Geeurickx E et al. Nat Commun 10:3288 (2019).

anti-Met (c-Met) antibody (ab51067, https://www.abcam.com/products/primary-antibodies/met-c-met-antibody-ep1454y-n-terminal-ab51067.html) has been validated in several experimental settings: WB with wild-type HAP1 cell lysate, HepG2, HEK-293, Hela and A459 cell lysate, mouse and rat thymus tissue lysate, mouse lung tissue lysate; IHC-P with human bladder carcinoma and clear cell kidney carcinoma tissue. This antibody has been referenced in 87 publications. Selected recent citations:
Wu X et al. J Exp Clin Cancer Res 40:70 (2021);
Li M et al. Cancer Manag Res 13:2235-2246 (2021);
Pereira PMR et al. J Nucl Med 62:366-371 (2021).

Anti-Cyanine Antibody (sc-166936, https://www.scbt.com/p/anti-cyanine-antibody-a-12). The family of cyanine dyes include Cy2, Cy3, Cy5, Cy7 and their derivatives. This antibody has been validated in western blot analysis of cyanine expression in Cy5-conjugated BSA. It has been referenced in 6 publications. Selected citations:
Lee H et al. J. Photochem. Photobiol. A Chem. 200: 438-444 (2008);
Wang Y et al. Chemphyschem 11: 889-896 (2010);
Mahmood T et al. J. Org. Chem. 75: 204-207 (2010)

Eukaryotic cell lines

Policy information about cell lines

Cell line source(s)	Mouse cell line MC26-LucF (carrying firefly luciferase) was received from Kenneth K. Tanabe Laboratory, Massachusetts General Hospital. Human cell lines PC-3 (ATCC CRL-1435) and RWPE1 (ATCC CRL-11609) are commercially available.
Authentication	The commercially available cell lines from ATCC have been thoroughly tested and authenticated using short tandem repeat profiling by the vendor. Mouse cell lines MC26-LucF (carrying firefly luciferase, from Kenneth K. Tanabe Laboratory, Massachusetts General Hospital) was previously validated upon receipt, selected references are:<br>Danino T et al., Sci Transl Med 7, 289, pp. 289ra84 (2015)<br>Kwon E et al., Nat Biomed Eng 1, 0054 (2017).<br>We also carefully examined the morphology of the cell line in culture and confirmed it with mouse-specific qPCR primer to confirm the expression of housekeeping genes ATCB and TBP.
Mycoplasma contamination	All cell lines were tested negative for mycoplasma contamination.
Commonly misidentified lines<br>(See ICLAC register)	This study did not involve commonly misidentified lines.

Animals and other organisms

Policy information about studies involving animals; ARRIVE guidelines recommended for reporting animal research

Laboratory animals	Female Balb/c (BALB/cAnNTac, 6-8 wks of age, Taconic Biosciences), female NCr nude (CrTac:NCr-Foxn1nu, 4-5 wks of age, Taconic Biosciences), female and male KrasLSL-G12D/+;Trp53fl/fl C57L/B6 (KP) mice (8-16 wks of age, gift from Tyler Jacks Laboratory, MIT) were used for experiments. Mice were maintained in the Koch Cancer Institute animal facility, with a 12-h light/12-h dark cycles (7am to 7pm), at ~18-23°C and ~50% humidity. Autoclaved water and standard chow diet were accessible at all times. NCr nude mice were housed in autoclaved cages and paper bedding in a room with other immunodeficient-only animals.
Wild animals	This study did not involve wild animals.
Field-collected samples	This study did not involve field-collected samples.
Ethics oversight	All animal studies were approved by the Massachusetts Institute of Technology (MIT) committee on animal care (MIT protocol 0420-023-23 & 0220-010-23). All experiments were conducted in compliance with institutional and national guidelines and supervised by Division of Comparative Medicine (DCM) of MIT staff.

Note that full information on the approval of the study protocol must also be provided in the manuscript.
